# Microbial-driven nature-based solutions for environmental antimicrobial resistance and emerging contaminants: mechanisms, platform trade-offs, and decision framework

**DOI:** 10.3389/fmicb.2026.1804764

**Published:** 2026-05-19

**Authors:** Atin Kumar, Nilotpal Das, Sandeep Gawdiya, Rohit Kumar, Pradeepto Pal, Sharad Sachan, Pritam Ghosh, Hanuman Singh Jatav, Caleb Ayomide Babatunde

**Affiliations:** 1 School of Agriculture, Uttaranchal University, Dehradun, Uttarakhand, India; 2 School of Agriculture, Galgotias University, Greater Noida, Uttar Pradesh, India; 3 Faculty of Agricultural Sciences, GLA University, Mathura, Uttar Pradesh, India; 4 College of Horticulture, Central Agricultural University, Imphal, Manipur, India; 5 Department of Agricultural Economics and Extension, School of Agriculture, Lovely Professional University, Phagwara, India; 6 Department of Geography, Hijli College, Kharagpur, India; 7 Department of Soil Science and Agricultural Chemistry, Sri Karan Narendra Agriculture University, Jobner, Rajasthan, India; 8 Pedology and Land Use Research Group, Department of Soil Resources and Environmental Management, Ekiti State University, Ado Ekiti, Nigeria

**Keywords:** antimicrobial resistance, bioremediation, emerging contaminants, environmental sustainability, microbial consortia, nature-based solutions

## Abstract

**Introduction:**

Antimicrobial resistance (AMR) and emerging contaminants (ECs), including pharmaceuticals, personal care products, microplastics, and endocrine-disrupting chemicals, pose interconnected threats to environmental and human health. Nature-based solutions (NbS) have emerged as sustainable and cost-effective approaches for mitigating these challenges through ecosystem-driven processes.

**Methods:**

This review follows a PRISMA-guided narrative-systematic synthesis of literature published between 2000 and 2024, using data sources including Scopus, Web of Science, and PubMed. The analysis integrates evidence on microbial mechanisms, NbS platform performance, and environmental AMR-EC interactions.

**Results:**

The synthesis highlights that microbial-driven NbS exploit metabolic diversity, functional plasticity, and plant-microbe interactions to degrade, transform, immobilize, or eliminate contaminants in soil, water, and wastewater systems. Advances in microbial ecology, synthetic biology, and omics approaches have enabled the design of functional microbial consortia capable of targeting antibiotic residues, resistance genes, and recalcitrant pollutants. NbS platforms such as constructed wetlands, rhizosphere-based systems, biofilters, and microbial electrochemical technologies demonstrate variable performance influenced by microbial diversity, redox processes, and system design. However, trade-offs exist, including the potential for microbial biofilms to act as reservoirs of antibiotic resistance genes.

**Discussion:**

Despite their potential, microbial-driven NbS face challenges related to scalability, long-term performance, ecological risks, and regulatory acceptance. This review proposes a microbial NbS decision framework linking environmental sources, microbial mechanisms, platform design, and monitoring indicators to support sustainable and risk-aware implementation. Overall, the effectiveness of NbS depends on optimizing microbial functional diversity, system design, and resistance suppression strategies to ensure long-term environmental and public health benefits.

## 1 Introduction

**Figure 1:**
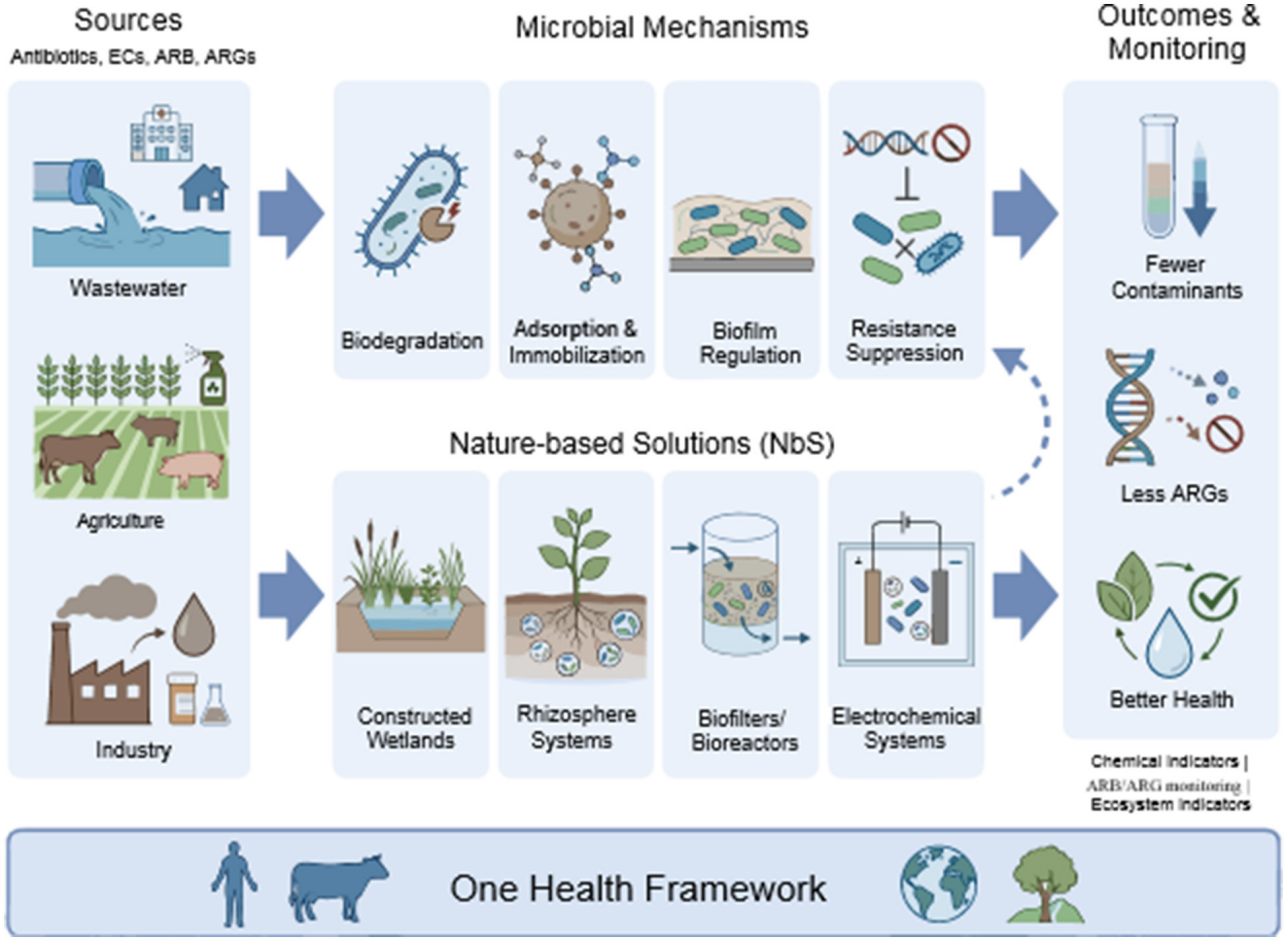
Conceptual framework illustrating microbial-driven nature-based solutions (NbS) for mitigating antimicrobial resistance (AMR) and emerging contaminants (ECs), linking environmental sources, microbial mechanisms, NbS platforms, and monitoring indicators within a One Health framework. Environmental contamination sources generate selective pressures that shape microbial communities in soil and aquatic systems. These microbial processes operate within NbS platforms such as constructed wetlands, rhizosphere-based systems, biofilters, and microbial electrochemical technologies. Integrated monitoring indicators—including chemical contaminant levels, antibiotic-resistant bacteria (ARB), and resistance genes (ARGs)—support adaptive management and evaluation of contaminant removal, resistance suppression, and ecosystem resilience within a One Health framework (Source: Created by the authors).

Antimicrobial resistance (AMR) and emerging contaminants (ECs) are increasingly recognized as interconnected environmental challenges driven by the release of antibiotics, pharmaceuticals, and other pollutants into soil, aquatic, and wastewater systems. AMR is already estimated to cause 1.27 million deaths annually, and has been linked to about 5 million deaths globally, and it is projected that the number of deaths caused by drug resistant infections could reach up to 10 million deaths per year in 2050, unless current trends are altered, overtaking cancer as a leading cause of mortality (Hamers et al., 2023; Nazir et al., 2025). It is disproportional in low and middle-income countries, which have weak sanitation systems, have limited diagnostic capacity, use antimicrobials irresponsibly, and have limited access to high-quality medicines and protection against infections, increasing exposure and vulnerability (Hamers et al., 2023; Eke and Cua, 2025). Simultaneously, the popular application of ECs, such as antibiotic residues, biocides, pesticides, industrial chemicals, and pharmaceutical byproducts, into soils, surface waters, groundwater, and food systems is transforming the microbial communities and is selecting resistant bacteria and resistance genes in the majority of cases outside the clinical practices (Qiu et al., 2022; La Rosa et al., 2025). Wastewater treatment plants, aquaculture systems, livestock operations, and agri-food chains serve as major sources of antibiotics, antibiotic-resistant bacteria (ARB), and antibiotic resistance genes (ARGs). These environments also facilitate horizontal gene transfer and co-selection with other pollutants, including heavy metals and pesticides (Qiu et al., 2022; La Rosa et al., 2025; Zackariah et al., 2025). These interactions underscore the pivotal role of environmental systems in shaping antimicrobial resistance dynamics and highlight the need for mitigation strategies that extend beyond clinical and pharmaceutical settings. Viewed through the prism of One Health and planetary health, AMR and ECs are therefore more aptly seen as the emergent characteristics of coupled social-ecological systems, which are fueled by intensive food production, urbanization, climate change, global trade, and unfair access to sanitation and health care (Horvat and Kovačević, 2025; Astbury et al., 2025; Eke and Cua, 2025). In this framework, the concept of Nature-Based Solutions (NbS), which is an intervention designed to secure, control, or reinstate the ecosystem to respond to societal pressures, has become the most popular means of reducing pollution loads, stabilizing ecosystems, and increasing their resilience. The recent research on constructed wetlands, riparian buffers, and other ecological treatment systems has shown that NbS is capable of attaining significant levels of reductions in environmental AMR and generating effluents that can be safely reused (La Rosa et al., 2025). NbS have evolved from conservation-oriented ecosystem service concepts toward problem-specific, process-driven designs based on the concept of utilizing ecological processes, including sorption, photodegradation, plant uptake, and microbially mediated biotransformation, to achieve water, food, climate, and health goals in an integrated fashion. Microorganisms play a key role in the functioning of these systems: they help to achieve degradation, transformation, and immobilization of ECs, regulate biofilm formation and community assembly, and affect whether resistance genes in changing physicochemical conditions are amplified or suppressed (La Rosa et al., 2025; Davis et al., 2023). To enhance EC removal, disrupt resistance transmission, and maintain ecological equilibrium, microbial-driven NbS specifically designs microbial ecology by hydrological design, selection of substrates, plant-microbe interactions, redox gradient, and community engineering to avoid configurations that recreate new hotspots of ARG exchange. These strategies do not substitute antimicrobial stewardship and technological advancements in the field of diagnostics and therapeutics; instead, they create an essential gap in prevention strategies in the environment and upstream prevention approaches that have been identified as a deficiency in global AMR assessments (La Rosa et al., 2025; Hamers et al., 2023; Davis et al., 2023). Against this background, microbial-driven nature-based solutions (NbS) are increasingly recognized as promising interventions for mitigating antimicrobial resistance (AMR) and emerging contaminants (ECs) in environmental systems. However, existing studies and reviews largely address individual contaminants, microbial processes, or NbS platforms in isolation, with limited integration of microbial mechanisms, platform-specific trade-offs, resistance risks, and performance evaluation within a unified decision-making framework. In particular, the dual role of microbial communities—as both agents of contaminant attenuation and potential reservoirs or amplifiers of resistance—remains insufficiently synthesized across systems and scales. To address these gaps, this review advances an integrative, systems-level perspective that links environmental sources and selective pressures, microbial mechanisms governing contaminant transformation and resistance modulation, NbS platform design choices, multi-level monitoring indicators, and governance considerations under a One Health paradigm. This synthesis is operationalized through a microbial NbS decision framework for AMR–EC mitigation (Figure 1), which provides a structured basis for selecting, designing, and managing NbS technologies while explicitly balancing remediation efficiency with resistance suppression and long-term sustainability. Figure 1 provides a unifying conceptual scaffold for the subsequent sections, linking microbial mechanisms (Section 5), NbS platform performance (Section 6), technological innovations (Section 7), and monitoring and governance considerations (Section 8).

Within this framework, the review examines (i) environmental sources and pathways of AMR and ECs, (ii) microbial mechanisms and NbS platform performance, (iii) monitoring and uncertainty-aware evaluation approaches, and (iv) future research and policy priorities.

### 1.1 Scope and boundaries of this review

This review focuses specifically on the role of microbial mechanisms within nature-based solutions (NbS) for mitigating antimicrobial resistance (AMR) and emerging contaminants (ECs) in environmental systems. The central emphasis is placed on microbial-driven processes, including biodegradation, biotransformation, adsorption and immobilization, biofilm-mediated regulation, and microbial interactions with plants and engineered substrates that underpin the performance and resilience of NbS across soil, aquatic, and wastewater environments.

To maintain analytical clarity and depth, clinical antimicrobial resistance, pharmaceutical drug discovery, vaccine development, and human or veterinary therapeutic interventions are outside the scope of this review. While the human health implications of environmental AMR are acknowledged through a One Health perspective, the analysis is confined to environmental reservoirs, transmission pathways, and mitigation strategies.

Artificial intelligence (AI), machine learning (ML), and digital technologies are discussed only in the context of monitoring, modeling, and optimization of microbial NbS, such as performance assessment, decision-support systems, and uncertainty-aware management. These technologies are not treated as primary remediation tools but as enabling frameworks that enhance system understanding and operational efficiency.

Policy, governance, and sustainable development considerations are included only where they directly influence the implementation, regulation, and long-term effectiveness of environmental AMR mitigation and NbS deployment. Broader discussions on global health governance or pharmaceutical policy are not addressed unless they intersect with environmental contamination pathways, wastewater management, or ecosystem-based interventions.

By clearly defining these boundaries, the review aims to provide a focused, mechanistically grounded, and systems-level synthesis of microbial innovations in NbS, while avoiding dilution across unrelated clinical or technological domains.

Environmental contaminants are treated not only as stressors but also as key drivers of microbial selection pressure, resistance propagation, and ecological feedback mechanisms within nature-based treatment systems.

## 2 Methods

### 2.1 Review design and reporting framework

The literature search used structured Boolean search strings combining keywords related to antimicrobial resistance, emerging contaminants, microbial processes, and nature-based solutions. An example of the search string applied in Scopus and Web of Science was: (“antimicrobial resistance” OR “antibiotic resistance” OR “ARGs”) AND (“emerging contaminants” OR pharmaceuticals OR pesticides OR microplastics) AND (“nature-based solutions” OR “constructed wetlands” OR “rhizosphere remediation” OR “biofilters” OR “microbial electrochemical systems”) AND (“biodegradation” OR “biofilm” OR “biotransformation” OR “microbial remediation”). Similar search strings were adapted for each database.

The literature search covered studies published between January 2000 and December 2024, capturing the rapid expansion of research on antimicrobial resistance, emerging contaminants, and microbial remediation technologies.

Only articles published in English were considered to ensure consistency in interpretation and methodological comparison.

**Figure 2:**
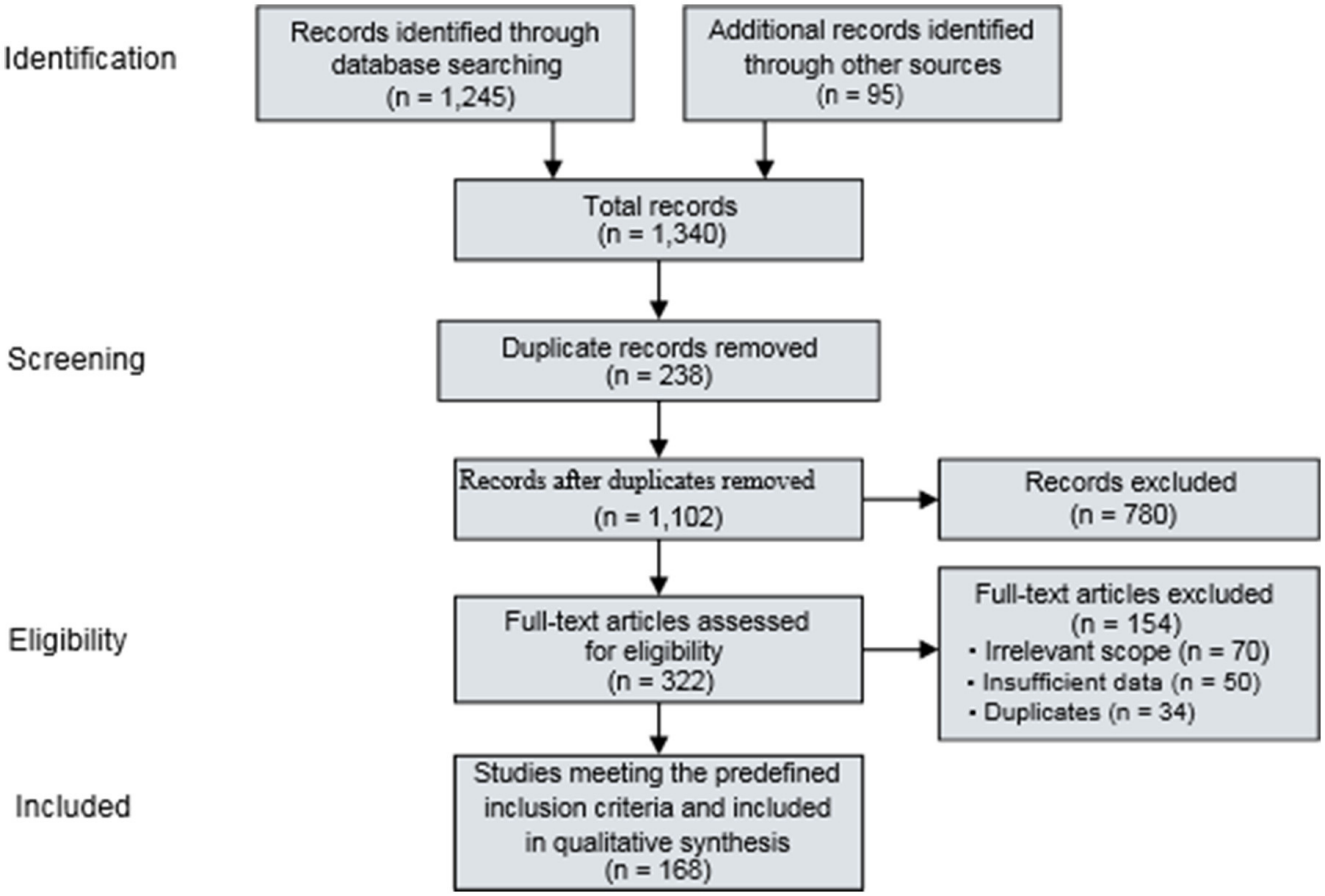
PRISMA 2020 flow diagram illustrating the identification, screening, eligibility assessment, and inclusion of studies for the narrative–systematic hybrid review on microbial-driven nature-based solutions for antimicrobial resistance and emerging contaminants (Source: Created by the authors).

This review followed the Preferred Reporting Items for Systematic Reviews and Meta-Analyses (PRISMA 2020) guidelines to ensure transparency and reproducibility in the literature identification, screening, eligibility assessment, and synthesis processes. The overall study selection workflow is summarized using a PRISMA 2020 flow diagram (Figure 2).

A narrative–systematic hybrid synthesis was selected **“because”** the objectives of this review required integration of mechanistic, platform-level, and monitoring evidence **“AND”** involved substantial heterogeneity in study designs, outcome measures, and reporting of antimicrobial resistance indicators. Quantitative synthesis was considered appropriate **“only if”** sufficiently comparable effect measures and standardized resistance metrics were available across studies, which was not observed during eligibility assessment.

The review protocol followed PRISMA 2020 reporting recommendations for systematic reviews addressing complex environmental interventions.

### 2.2 Literature search strategy

A systematic literature search was conducted in Scopus, Web of Science, and PubMed, using combinations of keywords related to AMR, emerging contaminants, microbial processes, and nature-based solutions, including constructed wetlands, rhizosphere systems, biofilters, and microbial electrochemical technologies.

The database search identified 1,245 records, and an additional 95 records were retrieved through manual reference screening of relevant articles, yielding a total of 1,340 records prior to duplicate removal. The final literature search was conducted in December 2024.

### 2.3 Study screening and eligibility

After removing 238 duplicates, 1,102 unique records were screened based on titles and abstracts. Of these, 780 records were excluded for being outside the scope of the review, leaving 322 articles for full-text assessment.

Full-text screening resulted in the exclusion of 154 articles due to a clinical-only AMR or drug discovery focus (*n* = 53), absence of a microbial component (*n* = 22), or lack of relevance to nature-based solutions (*n* = 79). The literature identification, screening, eligibility assessment, and final inclusion process followed in this review are illustrated in the PRISMA 2020 flow diagram (Figure 2).

Studies were included if they (i) examined antimicrobial resistance, antibiotic resistance genes, or emerging contaminants in environmental systems; (ii) investigated microbial mechanisms such as biodegradation, biofilm processes, or biotransformation; (iii) evaluated nature-based solutions or related microbial remediation systems including constructed wetlands, rhizosphere systems, biofilters, or microbial electrochemical technologies; and (iv) reported experimental or observational evidence related to contaminant removal or resistance mitigation.

The title and abstract screening process was conducted independently by two reviewers. Discrepancies were resolved through discussion and consensus. Full-text screening and data extraction were subsequently performed using predefined inclusion criteria.

Full-text articles were excluded when they focused solely on clinical antimicrobial resistance, lacked a microbial mechanism relevant to environmental processes, or were not related to nature-based treatment systems.

### 2.4 Study quality assessment

To ensure the reliability of the evidence included in the review, the methodological quality of selected studies was evaluated using criteria commonly applied in environmental systematic reviews. Each study was assessed based on the clarity of experimental design, description of microbial processes, contaminant measurement methodology, and reporting of removal efficiency or resistance mitigation outcomes. Studies lacking sufficient methodological detail or quantitative evidence were excluded from the synthesis. The quality appraisal results were incorporated into the PRISMA screening dataset to maintain transparency and reproducibility.

A quality assessment score was incorporated into the PRISMA screening dataset to ensure that only studies meeting minimum methodological standards were included in the final synthesis.

### 2.5 Data synthesis

Following the eligibility assessment, a total of 168 studies met the predefined inclusion criteria and were included in the qualitative synthesis. These studies formed the evidence base for the narrative–systematic integration of microbial mechanisms, platform performance, and resistance dynamics. For each included study, key information was extracted, including study design, environmental system type, contaminant categories investigated, microbial mechanisms involved, reported contaminant removal performance, and antimicrobial resistance indicators such as antibiotic-resistant bacteria (ARB) and antibiotic resistance genes (ARGs).

The synthesis adopted a narrative–systematic approach, integrating evidence across heterogeneous environmental systems including soils, aquatic environments, wastewater treatment systems, and nature-based treatment platforms. Because the reviewed studies varied widely in experimental design, environmental matrices, contaminant types, and resistance monitoring methods, a quantitative meta-analysis was not considered appropriate. Instead, the analysis focused on identifying recurring mechanistic patterns, platform-specific performance trends, and cross-system interactions linking microbial processes, nature-based solutions (NbS), and antimicrobial resistance dynamics.

The included studies were thematically analyzed to synthesize evidence across four main dimensions:

1. environmental sources and pathways of antimicrobial resistance and emerging contaminants;
2. microbial mechanisms governing contaminant transformation, immobilization, and resistance modulation;
3. performance and trade-offs of different NbS platforms;
  and
4. monitoring indicators and governance considerations within a One Health framework.

Because the objective of this review is to provide a conceptual and mechanism-oriented synthesis rather than a comprehensive catalog of all screened studies, only the most representative and influential studies supporting specific mechanisms, technologies, and monitoring approaches are explicitly cited in the manuscript. Consequently, the reference list includes 97 key references, while the broader set of 168 eligible studies informed the comparative analysis, conceptual synthesis, and framework development presented in this review.

The synthesized evidence directly informed the conceptual microbial NbS decision framework (Figure 1) and the comparative platform analysis (Table 1). This synthesis approach emphasizes integration of microbial mechanisms, system design considerations, and resistance mitigation strategies across environmental contexts, consistent with PRISMA-2020 guidance for reviews addressing complex environmental interventions with heterogeneous evidence bases.

**Table 1:** Comparative performance of microbial-driven nature-based solutions (NbS) for the mitigation of antibiotics and antimicrobial resistance (AMR) indicators.

Platform	Target contaminants	Reported ARG/ARB trends (qualitative)	Typical study scale	Commonly reported limitations	References
Constructed wetlands (CWs; surface, subsurface, hybrid)	Municipal/domestic wastewater; some agri-/aquaculture effluents; mainly antibiotics (sulfonamides, macrolides, tetracyclines, fluoroquinolones) plus nutrients and conventional pollutants	ARGs/ARB: mesocosm and field CWs generally report ~0.5–3 log reductions for common genes (sul, tet, erm, intI1) and substantial ARB inactivation (up to ~99% ARB removal in some lab HSSF systems); performance varies with configuration (HSSF vs. VSSF vs. surface flow), plant species, loading, and maturity, and ARG reduction is often incomplete with possible persistence in substrates and biofilms	Wide range from bench-scale micro-/mesocosms and lab pilots to full-scale and field-scale tertiary wetlands	Reported limitations include variable seasonal performance, sensitivity to loading and HRT, lower removal for certain antibiotics (notably some sulfonamides/macrolides), long start-up times, substrate saturation and biomass management issues, and only partial/heterogeneous ARG suppression with potential long-term accumulation in sediments	McCorquodale-Bauer et al., 2023; Liu et al., 2019; Zhang R.-M. et al., 2025; Sabri et al., 2021; Pastor-Lopez et al., 2024; Chen et al., 2016; Aslam et al., 2025; Yuan et al., 2025; Cusumano et al., 2025
Rhizosphere/phytomicrobial systems (phytoremediation in soils and aquatic matrices, including intercropping and biochar-amended fields)	Soil and rhizosphere contaminated with antibiotics, heavy metals and ARGs; mainly sulfonamides, tetracyclines and mixed antibiotics in agricultural soils and irrigation waters	ARGs: responses appear mixed; some soil phytoremediation systems report increased ARG abundance under sole cropping, while intercropping or biochar amendment can reduce ARGs in rhizosphere or plant endophytes by roughly 20–>90% (≈1–2 log in endophytes) relative to controls. Overall, reported evidence suggests partial mitigation in plant tissues and rhizosphere, but limited or inconsistent reductions in bulk soil, with MGEs remaining important in ARG propagation	Mainly lab and mesocosm soil/greenhouse studies, plus some field-scale trials assessing biochar-amended crop systems and ARGs in soil–plant compartments	Frequently cited limitations include variable efficiency across plant species and soils, risk of phytotoxicity, limited knowledge of transformation products, incomplete control of ARGs in bulk soil, and uncertainties around long-term stability and safe management of contaminated biomass; performance often depends strongly on root architecture and microbiome composition	McCorquodale-Bauer et al., 2023; Cui et al., 2021; Zhou et al., 2024; Cusumano et al., 2025
Biofilters/MBR/MBBR and other biofilm-based reactors	Municipal and industrial (including pharmaceutical) wastewaters; antibiotics across multiple classes (β-lactams, macrolides, sulfonamides, quinolones, tetracyclines) plus broader pharmaceuticals and conventional pollutants	ARGs/ARB: full-scale MBRs typically show substantial ARB removal (often >2–3 log) and notable but incomplete ARG reduction (roughly 1–several log, with some genes persisting in permeate or as cell-free DNA); membrane fouling layers and biofilms can enhance retention and mitigate dissemination (Wang et al., 2023, 2020; Le et al., 2018). Biofilm reactors and MBBR-type systems display more variable trends, with some studies reporting concurrent PPCP and ARG reductions, and others noting ARG enrichment within biofilms or effluents depending on carriers, loading and redox conditions	Predominantly pilot- and full-scale municipal and pharmaceutical WWTPs for MBR/AnMBR, plus lab/pilot MBBR and other carrier-based biofilm systems; some systematic/bibliometric and process reviews	Reported limitations include energy demand and operational complexity for MBRs, membrane fouling, compound-specific recalcitrance, incomplete ARG removal with effluent cell-free ARGs considered a residual risk, and in some MBBR/biofilm designs, potential ARG enrichment or dependence on careful control of carrier fill, biofilm thickness and DO	Wang et al., 2023; Nasrollahi et al., 2022; Saidulu et al., 2021; Mishra et al., 2023; Wang et al., 2020; Le et al., 2018
Microbial electrochemical technologies (METs/BES, including potential-difference-enhanced MABR-type systems)	Antibiotics and other emerging pharmaceuticals in synthetic and real wastewaters; commonly sulfonamides (e.g., sulfamethoxazole), macrolides (erythromycin), tetracyclines, and veterinary antibiotics; sometimes co-contaminants such as nutrients	ARGs: evidence remains relatively limited but suggests that well-controlled potential gradients can suppress specific resistance genes in biofilms (e.g., sul1 and sul2 reductions on the order of ≥1 log linked to inactivation of key host genera). At the same time, reviews of electrochemical stimulation indicate that sub-optimal or low-intensity fields may enhance horizontal and vertical gene transfer, so METs are often described as having a dual or “double-edged” influence on ARGs that is highly sensitive to operating conditions	To date, most MET studies addressing antibiotics and ARGs are lab-scale reactors (MFCs, bio-cathodes, electro-Fenton and potential-difference-enhanced MABR configurations), with relatively few pilot- or field-scale demonstrations for antibiotic-laden waters	Commonly reported limitations include scale-up challenges, energy and materials costs (electrodes, catalysts), sensitivity of performance and ARG outcomes to current density and redox conditions, limited long-term and field data on ARG dynamics, and concerns that poorly controlled micro-electric fields could unintentionally promote ARG transfer despite good contaminant removal	Zhang H. et al., 2025

## 3 Antimicrobial resistance and emerging contaminants: environmental perspective

### 3.1 Sources and pathways

Antimicrobial resistance and emerging contaminants primarily arise from intensive antibiotic use and complex waste streams across agricultural, aquaculture, healthcare, and industrial sectors (Do et al., 2023). Collectively, healthcare facilities, pharmaceutical industries, livestock and aquaculture systems, and municipal wastewater infrastructures act as interconnected sources that introduce antibiotics, resistant microorganisms, and resistance genes into environmental compartments, where they persist and disseminate through complex soil–water–sediment pathways. Wastewater treatment plants (WWTPs) and manure management systems do not eliminate antibiotics and ARGs and, in most cases, often act as amplification and transfer points, discharging contaminated effluents and biosolids into rivers, lakes, agricultural soils, recharge areas (groundwater), and sediments (La Rosa et al., 2025; Cheung et al., 2025; Maldonado et al., 2025; Do et al., 2023). Hospital effluents represent a particularly concentrated source of antimicrobial resistance drivers, including high loads of antibiotics, disinfectants, antibiotic-resistant bacteria (ARB), and resistance genes (ARGs). These waste streams often enter municipal wastewater systems without adequate pre-treatment, contributing to elevated selective pressure and facilitating the dissemination of resistance determinants in downstream aquatic and soil environments. Upon entry into the environment, contaminants are distributed via soil leaching, overland flow, groundwater infiltration, sediment deposition, resuspension, and bioaerosols and entrap AMR and co-occurring pollutants into interconnected compartments on land and in the water (Deza-Cruz et al., 2025; Stanton et al., 2022; Perković et al., 2022). Table 2 synthesizes the major source sectors of antimicrobial resistance and emerging contaminants, highlighting dominant environmental pathways and their implications for resistance dissemination.

**Table 2:** Key sources–pathways linkages for AMR and ECs.

Source sector	Main contaminants	Dominant environmental pathways	References
Livestock & poultry	Antibiotics, ARB, ARGs, manure-borne ECs	Manure application → soil → runoff → surface water/groundwater; dust and bioaerosols	Hanna et al., 2023; Enshaie et al., 2025; Perković et al., 2022
Aquaculture	In-pond antibiotics, ARB, ARGs	Pond effluents → rivers, estuaries, coastal/mangrove sediments	Zheng et al., 2021; Lertcanawanichakul et al., 2025; Mohammed et al., 2025
Human healthcare & households	Antibiotic residues, ARB/ARGs, PPCPs	Sewer → WWTPs → effluents, sludge → rivers, soils, sediments, groundwater	La Rosa et al., 2025; Cheung et al., 2025; Hanna et al., 2023
Pharmaceutical & other industry	High-strength antibiotic waste, industrial chemicals	Untreated/insufficiently treated wastewater and solid waste → local water bodies, sediments	La Rosa et al., 2025; Hanna et al., 2023; Wu et al., 2025
Agriculture & urban land	Pesticides, PPCPs, microplastics, metals	Runoff, leaching, wind erosion → soil, surface water, groundwater	Deza-Cruz et al., 2025; Egbuna et al., 2021; Perković et al., 2022

### 3.2 Types of emerging contaminants

Emerging contaminants relevant to antimicrobial resistance are increasingly recognized for their co-occurrence, persistence, and capacity to exert selective pressure on microbial communities. Rather than acting in isolation, emerging contaminants co-occur in complex mixtures that promote co-selection and resistance development. Common antibiotic classes contributing to resistance selection in environmental systems include fluoroquinolones, sulfonamides, tetracyclines, β-lactams, and macrolides, which are frequently detected in wastewater and agricultural environments.

### 3.3 Environmental and ecological risks

The accumulation of antibiotics, ECs, and high microbial biomass in environmental matrices spurs the dissemination and propagation of ARGs. Such co-occurrence complicates risk assessment and remediation, as contaminant mixtures often exert synergistic or antagonistic effects on microbial selection and ecosystem functioning. Many receiving waters located downstream of wastewater treatment plants, hospitals, and intensive agricultural systems have been reported to exceed predicted no-effect concentrations for antibiotic resistance selection. These exceedances are particularly documented in rivers, sediments, and wastewater-impacted surface waters where antibiotic residues and resistance determinants accumulate (La Rosa et al., 2025; Zheng et al., 2021; Hanna et al., 2023). Horizontal transfer of ARGs between environmental and human/animal associated strains, between environmental and human/animal environments, and within soils, sediments, and water systems, happens through mobile genetic parts, including integrons and plasmids, transforming these environments into resistance long term reservoirs and mixing arenas (Zheng et al., 2021; Stanton et al., 2022; Perković et al., 2022). These stresses transform microbial diversity and ecosystem processes, changing community structure, nutrient cycling and critical processes, including the transformation of nitrogen and transformations of organic matter, greenhouse gas fluxes, and have been reported to change the community structure, nutrient cycling and changes in key processes in estuaries, coastal regions, mangroves and agricultural soils (Deza-Cruz et al., 2025; Zheng et al., 2021; Horvat and Kovačević, 2025; Perković et al., 2022). In humans and animals, AMR and ECs of environmental contamination form a variety of exposure pathways: drinking water, irrigation, food crops, aquaculture products, recreational waters, inhalation of dust and bioaerosols, which result in colonization or infection with resistant pathogens, diminished efficacy of treatment, and possible long-term effects of endocrine disruption and chemical toxicity (Horvat and Kovačević, 2025; Stanton et al., 2022; Egbuna et al., 2021). In addition, certain persistent contaminants and associated resistance determinants may undergo trophic transfer and biomagnification across food chains, potentially increasing exposure risks in higher organisms and amplifying long-term ecological and human health impacts. Although the AMR burden, and ecological degradation in general, caused by unregulated environmental contamination are not yet quantitatively estimated, there is a growing body of evidence to support the claim that this issue has a significant role in the global AMR burden and the overall ecological degradation, underscoring the need to implement integrated One Health and planetary health responses (La Rosa et al., 2025; Horvat and Kovačević, 2025; Stanton et al., 2022).

Collectively, these pathways and co-occurring risks indicate that antimicrobial resistance and emerging contaminants cannot be effectively mitigated through end-of-pipe treatment or clinical stewardship alone. Conventional wastewater treatment and single-technology interventions often reduce contaminant loads without addressing resistance selection, horizontal gene transfer, or long-term ecological persistence. Consequently, mitigation strategies must operate upstream within environmental systems, integrating ecological processes that simultaneously attenuate contaminants and suppress resistance dynamics. This need provides a direct rationale for nature-based solutions (NbS), which leverage ecosystem functions and microbial processes to intervene at critical environmental nodes where AMR and ECs emerge, persist, and propagate.

## 4 Nature-based solutions: concepts and framework

### 4.1 Definition and classification of NbS (nature-based solutions)

In response to the complex and interconnected environmental pathways described in Section 3, nature-based solutions (NbS) have emerged as a systems-level approach capable of addressing antimicrobial resistance (AMR) and emerging contaminants (ECs) at their ecological sources. NbS are defined as interventions inspired and supported by nature that protect, manage, or restore ecosystems while delivering environmental, societal, and economic co-benefits. In the context of AMR, NbS extend beyond pollutant removal to encompass microbial regulation, resistance suppression, and ecosystem resilience, positioning them as complementary strategies to conventional treatment technologies. NbS operate across a wide spectrum of ecosystems that are relatively pristine to those that are highly engineered. On one end are semi-natural systems in which the existing ecosystems (e.g., wetlands, rivers, forests) are either conserved or lightly managed to improve their regulating functions, e.g., flood attenuation or water purification. On the other end are engineered NbS, in which ecosystems are engineered or highly altered, typically in the form of constructed wetlands, bioretention cells, green roofs and living walls, using the principles of ecological engineering to provide certain ecosystem functions, such as stormwater management, contaminant removal or cooling in built environments (Almenar et al., 2020; Esraz-Ul-Zannat et al., 2024). In urban planning, NbS are commonly implemented through blue–green infrastructure as multifunctional mechanisms to control water, as well as to enhance microclimates and biodiversity, based on connections between green and aquatic features (parks, street trees, ponds, bioswales, river corridors) which are integrated with gray infrastructures (Esraz-Ul-Zannat et al., 2024). Green infrastructure and ecological engineering, therefore, fall within the NbS umbrella as design-based strategies that deliberately integrate biological elements and physical infrastructure to emulate, recover, or transform natural processes in the city and landscape.

In addition to ecosystem-based interventions, several engineered technologies that rely on microbial processes are frequently integrated with nature-based solutions. These include biofilters, membrane bioreactors (MBR), moving bed biofilm reactors (MBBR), and microbial electrochemical technologies (METs). Such systems are more accurately described as hybrid green–gray or ecologically engineered systems because they combine biological functions with engineered infrastructure. In this review, ecosystem-based platforms such as constructed wetlands and rhizosphere systems are treated as core NbS approaches, while biofilm-driven engineered systems are discussed as complementary technologies that extend the performance and controllability of NbS platforms.

### 4.2 Role of microorganisms in NbS

The performance of nature-based solutions (NbS) emerges from integrated ecosystem processes, including plant–microbe interactions, hydrological regulation, physicochemical transformations, and biodiversity-driven ecological functions. Within these coupled processes, microorganisms play a central role in nutrient cycling, contaminant biodegradation, biofilm formation, and resistance dynamics, thereby acting as key biological drivers of contaminant attenuation and ecosystem resilience (Wang et al., 2024; Kiruba N and Saeid, 2022). Under engineered NbS like constructed wetlands, biofilters and other types of ecological treatment systems, microbial consortia were found to form biofilms on immobile substances and plant roots that mediate on the decomposition of organic pollutants, the transformation of pharmaceuticals and industrial chemicals, denitrification and, in certain instances, the removal of antibiotic residues and their resistance determinants (Banerjee et al., 2023; Uy et al., 2025).

The microbial functional diversity, which is the range of metabolic properties and guilds provide redundancy and complementarity, enabling NbS to be able to perform at varying loading, climate stress, and disturbance (Banerjee et al., 2023; Wang et al., 2024; Uy et al., 2025). Communities are organized around microscale gradients in oxygen, redox potential, and nutrient availability caused by interactions between microbes, plants, hydrology and substrates and are used to establish sequential oxidation, reduction and sorption processes; an example of this is coupled aerobic anaerobic zones in wetlands (Banerjee et al., 2023; Uy et al., 2025; Esraz-Ul-Zannat et al., 2024). The control and regulation of these microbial environment feedbacks, by design, e.g., flow regime, substrate type, vegetation selection, and operational conditions, is then core to optimization of NbS to water purification, pollution control, and broader ecosystem service provision, including in the context of antimicrobial resistance, and more recently, emerging contaminants.

## 5 Microbial Mechanisms for mitigating antimicrobial resistance (AMR) and emerging contaminants (ECs)

### 5.1 Biodegradation and biotransformation

Microorganisms alleviate antibiotics and other emerging contaminants (ECs) by biodegradation and biotransformation mainly enzymatically (Anedda et al., 2023; Fang et al., 2023). Bacteria and fungi express oxidoreductases and other degradative enzymes that catalyze the biodegradation or biotransformation of antibiotics, pesticides, polycyclic aromatic hydrocarbons (PAHs), glyphosate, and plastic additives (Mu et al., 2023; Banerjee et al., 2023; Souza et al., 2025; Anedda et al., 2023). Key microbial groups involved in these processes include genera such as Pseudomonas, Bacillus, Acinetobacter, and Rhodococcus, which are widely reported for their metabolic versatility and ability to degrade a broad range of organic contaminants. Fungal taxa, including Aspergillus and Phanerochaete, also contribute significantly through ligninolytic enzyme systems capable of transforming recalcitrant compounds. E.g., glyphosate-degrading bacteria (e.g., Bacillus, Pseudomonas, Ochrobactrum) oxidize glyphosate through the aid of glyphosate oxidoreductase or C-P lyase to convert glyphosate into AMPA, sarcosine, glyoxylate, and inorganic phosphate, and most of them can use glyphosate as a source of C or P (Souza et al., 2025). Possessing similar catabolic routes, carcinogenic PAHs are trafficked through different mono and bio-oxygenases and channeled into the central metabolism by diverse bacteria and, in particular, fungi (Banerjee et al., 2023). Co-metabolism plays a role in complex systems, including mariculture microalgal-bacterial consortia, in the process of which antibiotics are accidentally transformed, and microorganisms oxidize primary substrates, and metabolic networks and redox gradients allow partial or complete detoxification (Gong et al., 2024; Mu et al., 2023). Further increased co-metabolic degradation is facilitated by the aging of microplastics and colonization by biofilms, which can act on rough and oxidized surfaces that increase microbial adhesion and enzyme activity (Sun et al., 2022; Luo et al., 2021). Overall, these examples illustrate how functional diversity and co-metabolism enable microbial communities to transform structurally diverse contaminants while can contribute to reducing resistance selection pressure under specific conditions. The principal microbial mechanisms underlying antimicrobial resistance and emerging contaminant attenuation across nature-based solutions are summarized in Table 3.

**Table 3:** Major microbial mechanisms against AMR and ECs.

Mechanism type	Key processes	Typical targets & systems	AMR/EC mitigation effects	References
Biodegradation & biotransformation	Oxidases, hydrolases, C–P lyase; co-metabolism in consortia	Antibiotics, PAHs, glyphosate, plastic additives; wetlands, bioreactors	Detoxifies organics, lowers antibiotic/EC concentrations, reduces selection for ARGs	Banerjee et al., 2023; Mu et al., 2023; Souza et al., 2025
Adsorption & immobilization	Biofilm EPS binding, biosorption, biomineralization, cell-wall ligands	Heavy metals, pesticides, antibiotics on biofilms, agro-waste, and soils	Converts mobile pollutants to less bioavailable forms; protects cells, limits exposure	Arias-Castro et al., 2025; Vasarevičius and Paliuliene, 2025; Manzoor et al., 2025
Biofilm-based vector modulation	Biofilm on MPs, biocarriers, WWTP biofilms	Microplastics, nutrients, and ARGs in wastewater	Concentrates pollutants; can both retain ARB/ARGs and facilitate removal if well-managed	Sun et al., 2022; La Rosa et al., 2025; Jagaba et al., 2021
Suppression of resistance development	Lower antibiotic levels, alternative stress relief, quorum/quenching, HGT interference	Wastewater, mariculture, soils, gut-like systems	Reduces selective pressure and horizontal gene transfer, constraining ARG spread	Shayista et al., 2024; Gong et al., 2024; La Rosa et al., 2025

In constructed wetlands and related nature-based systems, microbial communities play a critical role in the biodegradation of antibiotics and other emerging contaminants. Several studies have demonstrated that wetland-associated microbial consortia can transform or mineralize pharmaceutical residues through enzymatic processes, often in combination with adsorption and plant uptake mechanisms (Zheng et al., 2022; Lv et al., 2022; García et al., 2020). Biodegradation has been reported for multiple classes of pharmaceuticals, including fluoroquinolones, sulfonamides, tetracyclines, cephalosporins, and anti-inflammatory drugs such as ibuprofen, through microbial activity in wetland and rhizosphere environments (Alexandrino et al., 2017; Youssef et al., 2023).

Extracellular polymeric substances (EPS) produced by bacteria, algae, and fungi play an important role in the adsorption and immobilization of contaminants in environmental systems. EPS contain functional groups such as carboxyl, hydroxyl, phosphoryl, and amide groups that enable strong binding of heavy metals through ion exchange, complexation, surface adsorption, and precipitation mechanisms (Vandana et al., 2023; Pagliaccia et al., 2021; Priyadarshanee and Das, 2024). Experimental studies have reported high sorption capacities for metals such as Pb, Cu, Ni, and Zn, demonstrating the potential of EPS-mediated biosorption for contaminant removal and recovery in water treatment systems (Pagliaccia et al., 2021; Ajao et al., 2020). EPS matrices can also bind organic contaminants, including dyes, polycyclic aromatic hydrocarbons (PAHs), pesticides, and certain pharmaceuticals, contributing to pollutant sequestration and environmental bioremediation (Mahto et al., 2022; Vandana et al., 2023).

Nature-based treatment systems have also demonstrated the capacity to reduce antibiotic resistance genes (ARGs) in wastewater and environmental matrices. Constructed wetlands and related nature-based solutions commonly achieve 1–3 log reductions (approximately 90–99.9%) in ARG abundance depending on system configuration and gene type (Pastor-Lopez et al., 2024; Liu et al., 2019; Chen et al., 2016). Field-scale studies indicate that both surface-flow and subsurface-flow wetlands can effectively decrease ARG loads while simultaneously reducing antibiotic residues and associated ecotoxicological risks (Pastor-Lopez et al., 2024; García et al., 2020). Reviews and case studies further suggest that nature-based treatment systems consistently reduce antibiotic-resistant bacteria and resistance genes when used as tertiary polishing steps following conventional wastewater treatment (Hazra et al., 2022; Barancheshme and Munir, 2018; Gentile et al., 2024). However, although ARG abundances are substantially reduced, resistance determinants may remain detectable in treated effluents or biofilm communities, indicating that these systems mitigate rather than completely eliminate resistance dissemination (Liu et al., 2019; Abou-Kandil et al., 2020).

### 5.2 Adsorption, bioaccumulation, and immobilization

Microbial biofilms and extracellular polymeric substances (EPS) are mainly involved in the physical fixation of contaminants. Polysaccharide-rich, proteins and functional groups (carboxyl, hydroxyl, amino, phosphate) sorb antibiotics, metals and hydrophobic organics, restricting their several-fold diffusion into cells and the surrounding water (Gong et al., 2024; Sun et al., 2022; Jagaba et al., 2021). In a microalgal-bacterial system, antibiotic stress enhances the amount of EPS and protein abundance, which enhances adsorption barriers and lowers intracellular absorption of antibiotics, but does not remove extracellular transformations (Gong et al., 2024). High roughness and oxygenated groups on the age surfaces of microplastics lead to increased adsorption of pollutants and dense biofilm formation on the surface, which consequently determines the binding, transformative, and trophic transfer of organic pollutants and related ARGs (Sun et al., 2022; Luo et al., 2021; Fang et al., 2023). These systems combine sorptive capacity with active biodegradation. Microorganisms immobilized on natural media like agro-industrial wastes, biochar, soil particles, and plant roots combine sorptive capacity with biodegradation: lignocellulosic wastes provide porosity and reactive group sorptive capacity to pesticides, and attached degraders (e.g., Acinetobacter, Bacillus, Pseudomonas) increase pesticide removal and cell protection against washout (Arias-Castro et al., 2025). Bacteria mediate biosorption, bioprecipitation, and biomineralization of Cd, Pb, and Cu in metal-contaminated soils, and transform mobile ions into more stable forms and significantly decrease bioavailable fractions (Vasarevičius and Paliuliene, 2025; Manzoor et al., 2025). Such adsorption- bioaccumulation- immobilization processes reduce the levels of free contaminants and minimize ecological exposure and stabilize the polluted media. In addition, siderophore production by microorganisms can influence contaminant dynamics by chelating metal ions, thereby affecting metal mobility, bioavailability, and co-selection processes that contribute to antimicrobial resistance development.

### 5.3 Resistance development suppression

Antimicrobial resistance can also be inhibited and contained by microbial processes. Common environmental reservoirs of resistance include bacteria such as Escherichia coli, *Klebsiella* spp., *Acinetobacter* spp., and *Enterococcus* spp., which are frequently detected in wastewater, soils, and aquatic systems and are known to harbor diverse antibiotic resistance genes. With the assistance of degrading antibiotics or adsorbing them strongly in EPS and sorbent matrix, the communities reduce sub-inhibitory concentrations that promote the selection of resistant strains and ARG enrichment in aquatic and soil environments (Gong et al., 2024; La Rosa et al., 2025; Mu et al., 2023). Enhanced EPS production during antibiotic exposure of mariculture microalgae-bacteria systems suppresses antibiotic uptake by cells, and anaerobic bacterial dominance combined with increased ARG distribution is also linked to reduced antibiotic uptake by cells (Gong et al., 2024). Properly designed biological treatment and nature-based systems can then reduce selective pressure, but the biofilms in WWTPs and pipes can become ARG reservoirs and HGT hotspots instead (La Rosa et al., 2025; Jagaba et al., 2021). Reducing resistance, also, can be done through horizontal gene transfer (HGT): disrupting biofilm structure, quorum sensing, as well as oxidative and chemical stresses can be used to reduce conjugation rates and plasmid transfer that increase in dense, stressed biofilms (Shayista et al., 2024; La Rosa et al., 2025). HGT is also indirectly limited by some microbial communities and plant-microbe associations, which have a high functional diversity, which buffer environmental changes and offer alternative metabolic escape routes (Shayista et al., 2024; Wang et al., 2024; Manzoor et al., 2025).

Microbial biofilms play a central role in contaminant attenuation within nature-based and engineered treatment systems. Biofilm communities enhance the degradation of pharmaceutical compounds by creating metabolically diverse microenvironments that support cooperative microbial metabolism. For example, studies have demonstrated that biofilm-associated microbial communities contribute to the transformation of antibiotics and other pharmaceutical residues in wastewater and nature-based treatment systems (Brienza et al., 2022; Bertrans-Tubau et al., 2024). However, biofilms may also act as ecological reservoirs for antibiotic resistance genes (ARGs), where dense microbial populations and enhanced horizontal gene transfer can promote ARG persistence and dissemination (Uruén et al., 2020).

## 6 Microbial innovations in nature-based solutions (NbS) platforms

The comparative trends summarized in Table 1 are conceptually synthesized within the NbS decision framework (Figure 1), which highlights cross-platform trade-offs between removal efficiency and resistance risks.

The microbial mechanisms outlined in Section 5 including biodegradation, adsorption and immobilization, biofilm-mediated regulation, and resistance suppression are expressed differently across NbS platforms depending on system design, redox structure, plant–microbe interactions, and operational conditions. Consequently, NbS platforms differ not only in contaminant removal efficiency but also in their potential to mitigate or inadvertently amplify antimicrobial resistance. The comparative synthesis presented below situates individual platforms within this mechanism–performance–risk framework.

### 6.1 Constructed wetlands

Constructed wetlands (CWs) are NbS that are composed of hydraulics, plants, and porous media, which form redox stratified niches that support the development of different microbial communities, which catalyze the process of contaminant removal. Root oxygen release in planted systems creates aerobic microzones that support nitrification and oxidative transformation of organic contaminants, while deeper anoxic zones promote denitrification and other reductive transformation pathways that contribute to nutrient removal and contaminant attenuation. In unplanted beds, contaminant removal relies more strongly on microbial biofilms attached to substrate media and on physical filtration processes, although these systems may show lower microbial functional diversity and resilience (Da Silva and Wolff, 2024; La Rosa et al., 2025). Vertical-horizontal flow CWs, which combine aerobic and anaerobic layers, culminating in hybrid vertical-horizontal flow systems which couple aerobic and anaerobic compartments, can achieve removal efficiencies exceeding 90% for specific antibiotics under optimized pilot-scale conditions through sorption, biodegradation, and photolysis, and can lower AMR indicators (ARB/ARGs) by as much as 98% in pilot-scale systems (irrigation reuse). Particularly, ARGs bound to particles or expressed in bacteria are also intercepted by microbial biofilms and rhizosphere consortia, and thus reduced in released effluents; nevertheless, residual mobile ARGs can still be present and pose a risk (La Rosa et al., 2025; Paul et al., 2025).

### 6.2 Rhizosphere-based and phytomicrobial systems

**Figure 3:**
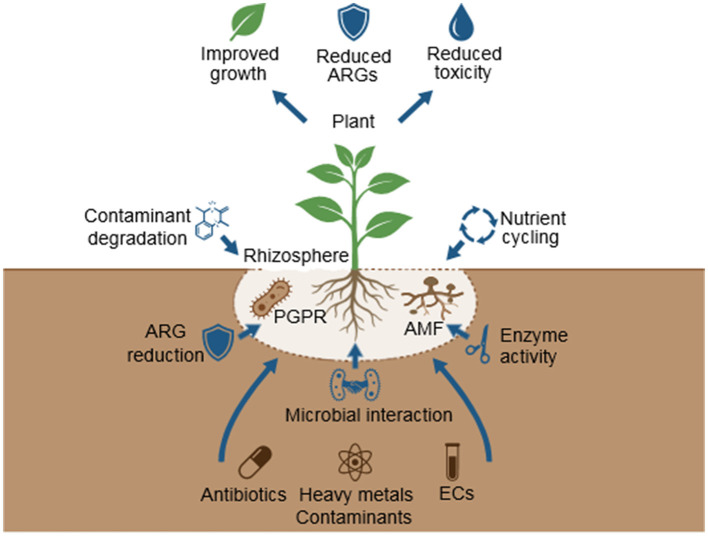
Phytomicrobial remediation processes in the rhizosphere support antimicrobial resistance (AMR) mitigation and emerging contaminant (EC) removal. Plant growth–promoting rhizobacteria (PGPR), arbuscular mycorrhizal fungi (AMF), and synthetic microbial communities (SynComs) interact with plant roots to regulate rhizosphere function. Root exudates drive microbial recruitment, biofilm formation, and enzymatic activity, enabling contaminant uptake, ARG attenuation, and inhibition of horizontal gene transfer. Together, these processes enhance contaminant removal while reducing resistance risks and supporting ecosystem services and plant health (Source: Created by the authors).

Rhizosphere-based NbS exploit plant–microbe interactions to enhance contaminant attenuation and system stability under field conditions. Plant growth-promoting rhizobacteria (PGPR) and arbuscular mycorrhizal fungi (AMF) promote degradation, immobilization, or uptake of metals, antibiotics, and organic micropollutants (Wang et al., 2024; Hnini et al., 2024). Root exudates (sugars, amino acids, organic acids, phenolics) are preferentially selective enrichment of functional microbiota, which supports co-metabolism and catabolic pathways to xenobiotics, and can also regulate the quorum sensing and biofilm formation of roots (Wang et al., 2024; Marín et al., 2021). Synthetic rhizosphere microbial communities (SynComs) have the power to design customized consortia with complementary properties, i.e., pesticide or antibiotic degraders and hormone-producing PGPR, to stabilize the performance of remediation and decrease the use of chemical inputs (Kiruba N and Saeid, 2022; Hnini et al., 2024; Marín et al., 2021). Recent experimental studies further support the critical role of rhizosphere-associated microorganisms in enhancing plant resilience under environmental stress conditions. For instance, halotolerant plant growth-promoting rhizobacteria (PGPR) such as *Agrobacterium tumefaciens, Bacillus subtilis*, and *Lysinibacillus fusiformis* have been shown to significantly improve seed germination, root and shoot growth, biomass accumulation, and chlorophyll content under saline stress conditions. These beneficial effects are primarily mediated through mechanisms such as phytohormone production (e.g., indole-3-acetic acid), ACC deaminase activity, nutrient solubilization, and modulation of rhizosphere microbial interactions. Such plant–microbe interactions not only enhance plant growth but also contribute to improved degradation and stabilization of contaminants in the rhizosphere, thereby strengthening the efficiency of phytomicrobial remediation systems (Mahmud et al., 2023). Furthermore, the enhancement of rhizosphere microbial activity can influence microbial community structure and reduce the persistence of contaminants and resistance determinants, supporting the role of phytomicrobial systems in sustainable environmental remediation. The key plant–microbe interactions, rhizosphere-driven microbial assembly processes, and functional roles of PGPR, AMF, and synthetic microbial consortia within phytomicrobial nature-based solutions are conceptually illustrated in Figure 3. Within the broader decision framework (Figure 1), these rhizosphere-level interactions represent a key mechanistic layer through which NbS platform design influences contaminant attenuation, resistance suppression, and long-term system resilience.

Plant–microbe interactions in the rhizosphere can significantly enhance the removal of pharmaceuticals and other emerging contaminants through synergistic microbial degradation and transformation processes. Rhizosphere microbial consortia stimulate contaminant breakdown through rhizodegradation, where pollutant-degrading bacteria utilize root exudates as co-substrates that activate catabolic pathways (Li et al., 2025; Cusumano et al., 2025; Kotrbová et al., 2025). Experimental studies and reviews have shown that microbial–phytoremediation systems can enhance the removal of antibiotics, polycyclic aromatic hydrocarbons (PAHs), dyes, and petroleum hydrocarbons compared with plant-only or microbe-only treatments (Das and Pandey, 2025; Kotoky et al., 2018; Wang et al., 2022). Root exudates such as phenolic compounds and flavonoids further stimulate rhizobacterial metabolic activity, thereby accelerating the degradation of aromatic pollutants and improving contaminant attenuation in rhizosphere environments (Kuzyanov et al., 2025; Kotoky et al., 2018).

### 6.3 Biofilters and bioreactors

The biofilters and bioreactors are engineered NbS where the fixed bed or fluidized microbial systems are optimized to treat at a high rate. In moving bed biofilm reactors (MBBR), membrane bioreactors (MBR), and hybrid moving bed membrane bioreactors (MBMBR), carrier-attached biofilms and/or suspended biomass are used to remove nutrients and a broad range of organic micropollutants through coupled adsorption and biotransformation (Saidulu et al., 2021; Elsayad et al., 2024; Ajaz et al., 2024). MBMBR is generally more effective than standalone MBR or MBBR in the removal of nutrients and organic materials, and as bioactive filters (e.g., BAC/GAC filters), a combination of adsorption of pharmaceuticals, personal care products, and pesticides onto the granular media with biofilms biodegrading parent compounds and transformation products is observed in particular when used as post-treatment to oxidative pretreatment (Saidulu et al., 2021; Ajaz et al., 2024). Municipal and industrial wastewater and agricultural runoff are progressively treated using such systems to minimize the release of antibiotics and ECs that spur the growth of ARG in discharge (La Rosa et al., 2025; Saidulu et al., 2021; Elsayad et al., 2024).

### 6.4 Electrochemical technologies with microbes

Microbial electrochemical technologies (METs) are a new type of bioelectrochemical NbS in which the electroactive bacteria on electrodes can catalyze the contaminant removal and produce or consume electrical current. Microbial fuel cells and other bioelectrochemical systems oxidize organic material at the anodes and reduce electron acceptors at the cathodes to permit the concomitant Chemical Oxygen Demand (COD) removal, energy harvesting, and, in certain configurations, the further conversion of antibiotics and other ECs (Gong et al., 2024; Mao et al., 2023). Electro-enhanced wetlands (alternatively known as electro-wetlands, METlands, CW MFC) add electrodes to planted beds to allow natural redox stratification and allow electroactive biofilms, and low, although constant, voltages generated by the systems may cause downstream electrochemical reactions that degrade antibiotics such as sulfamethoxazole without supplementary electricity (Gong et al., 2024; Mao et al., 2023). METs are also capable of changing the structure of a microbial community by enhancing redox gradients and activating individual electroactive guilds, which have potential opportunities to reduce resistance gene expression in NbS platforms, even though it remains an experimental approach (La Rosa et al., 2025; Gong et al., 2024; Mao et al., 2023).

## 7 Advances in microbial technologies supporting nature-based solutions (NbS)

### 7.1 Microbial consortia engineering

Nature-based solutions (NbS) increasingly rely on microbial consortia engineering, where, rather than the unmanaged black box communities, selectively assembled, steered, or designed communities are used. The soils, rhizospheres, and wetlands have high taxonomic and functional diversity in the form of bacteria, fungi, and archaea that cooperatively decompose food, cycle nutrients, suppress and eliminate pathogens, and help stabilize plant-soil interactions and increase resistance to disturbance (Wang et al., 2024; Marzouk et al., 2024). Engineered or synthetic communities (SynComs) represent a middle-out approach: consortia comprising dozens to hundreds of strains of defined composition are assembled either top down (to capture key taxa and functional capabilities of native community) or bottom up (to combine strains of complementary properties like phosphate solubilization, hormone production, or xenobiotic degradation). Other rhizosphere SynCom investigation demonstrates that functional strain mixing or inference by interaction networks can reconstitute significant beneficial functions of plants and accomplish mechanistic dissection of cooperation, rivalry, and cross-feeding (Marín et al., 2021). One of the main principles of the design is functional redundancy, i.e., several taxa having a similar role: redundancy helps NbS reactors, wetlands or rhizosphere systems to survive environmental shocks and invasion, as well as sustain the process rates (such as denitrification, contaminants degradation, etc.) when the composition of the community changes (Wang et al., 2024; Orr et al., 2025). Coexistence theory and ecological modeling are being considered to bring together consortia in a more systematic way that can be stable, with the intended functions in heterogeneous NbS environments (Orr et al., 2025). Recent technological advances that strengthen microbial functionality and stability within nature-based solutions are summarized in Table 4.

**Table 4:** Key advances linking microbial technologies and NbS.

Innovation area	Role in NbS platforms	Example advances	References
Consortia engineering (natural vs. designed)	Stabilize nutrient cycling, degradation, and plant health	SynComs mimicking core rhizosphere microbiomes; function-based strain selection	Wang et al., 2024; Marín et al., 2021; Marzouk et al., 2024
Omics & systems biology	Resolve “who does what, when, and how” in NbS	Metagenomics to map degraders/ARGs; multi-omics to link genes–pathways–metabolites	Corredor et al., 2024; Mishra et al., 2022; Di Carlo et al., 2022; Sherwani et al., 2025
Synthetic biology & bioaugmentation	Targeted degradation, value-added functions	Engineered strains for specific pollutants; bioorganic fertilizer consortia	Wang et al., 2024; Birgovan et al., 2025; Kiruba N and Saeid, 2022; Kurtoglu et al., 2024; Ahmad et al., 2021

### 7.2 Omics and systems biology approaches

Metagenomics, metatranscriptomics, and metabolomics have changed NbS designs of empirically designed systems to systems that can be rationally optimized. High-throughput metagenomics has become common in the profile of soil and water microbiomes, showing taxonomic composition, functional gene repertoires, and how they change as a result of pollution or remediation (Corredor et al., 2024; Mishra et al., 2022; Ahmad et al., 2021). Metagenomics can be used to demonstrate, in explosive-contaminated soils, the replenishment of diverse communities by less diverse but functionally enriched assemblages dominated by Proteobacteria and Actinobacteria bearing nitro-reducing and xenobiotic degradation genes, which provide biomarkers of monitoring and early intervention (Corredor et al., 2024). Shotgun metagenomics is becoming a widespread method to track resistomes in NbS treatment trains, and there are recommendations on sampling, sequencing depth, and normalization to ensure ARG profiles in different locations and times are similar (Mishra et al., 2022). Multi-omics is furthered by integrating metagenomics, metatranscriptomics, and metabolomics, key functional genes and pathways are connected to real activity and metabolite fluxes, and help to understand how microbial communities change pollutants and respond to hosts or plants (Birgovan et al., 2025; Di Carlo et al., 2022; Sherwani et al., 2025). As an illustration, integrated omics in bioremediation and food system microbiotechnologies would allow the identification of genes that mediate metabolic biotransformation pathways, aid in strain screening, and lead to more effective and stable bioprocesses that can valorize wastes and save synthetic inputs (Birgovan et al., 2025; Ahmad et al., 2021; Sherwani et al., 2025). NbS reactors under variable environmental conditions are increasingly being predicted with time series multi-omics with advanced machine learning or hybrid models to optimize engineered microbial processes (Cheng et al., 2025; Sherwani et al., 2025).

### 7.3 Synthetic biology and bioaugmentation

Synthetic biology and bioaugmentation are an expansion of NbS in terms of introducing or improving particular functions, but also have significant biosafety and ecological concerns. Targeted degradation of explosives, PAHs, plastics or newly emerging pollutants and transforming wastes into bioorganic fertilizers can be achieved by genetic and metabolic engineering to redesign bacteria, fungi and photosynthetic microorganisms to overexpress degradative enzymes, enhance stress tolerance, or synthesize bioactive compounds (Birgovan et al., 2025; Banerjee et al., 2023; Kiruba N and Saeid, 2022; Ahmad et al., 2021). Heterologous expression systems (e.g., Streptomyces chassis) and precision fermentation systems show how complicated biosynthetic pathways can be rewired or transplanted, and this knowledge can be applied in analogous strategies in the environmental context, e.g., pollutant-degrading or plant-protective metabolites (Birgovan et al., 2025; Lasch et al., 2025). As discussed in Section 7.1, the stability and resilience of nature-based solutions are strongly dependent on functional redundancy and cooperative interactions within microbial consortia, considerations that are particularly critical when introducing engineered strains or bioaugmentation strategies. The application of selected or engineered strains in bioaugmentation to explosives, emerging pollutants, and waste to fertilizer bioconversion, and frequently in consortia together with lignocellulolytic, chitinolytic, and plant growth-promoting microbes, is a preferred approach to ensure waste transformation and agronomic benefit (Banerjee et al., 2023; Kiruba N and Saeid, 2022; Ahmad et al., 2021). Nonetheless, the systematic reviews of synthetic biology ethics also raise the issues of dual use, control, unpredictability, and the ethical status of synthetic life and state that precautionary risk regulation is necessary, accompanied by clear communication and context-specific regulation (Kurtoglu et al., 2024). It has also been demonstrated that excessive use of individual patented strains can cause a decline in native biodiversity and restructure soil microbial networks, and that containment strategies, reduction of horizontal gene transfer, and design that rejuvenates or replenishes community diversity should be considered important (Corredor et al., 2024; Banerjee et al., 2023; Kurtoglu et al., 2024). Altogether, emerging technologies of engineered consortia, omics-directed systems biology, and synthetic biology offer potent tools to boost NbSs functions; however, their implementation should incorporate ecological theory and ethics to ensure ecosystem integrity and environmental sustainability.

## 8 Performance evaluation and monitoring

Performance monitoring in microbial NbS must be interpreted within a decision-support context that links contaminant removal, resistance dynamics, and system adaptation, as conceptualized in Figure 1. The application of nature-based and engineered systems to measure antimicrobial resistance (AMR) and emerging contaminants (ECs) requires the use of integrated indicators at chemical, microbiological, molecular, and ecosystem levels, which may require a One Health and planetary health approach to performance evaluation and monitoring (Horvat and Kovačević, 2025; Astbury et al., 2025).

### 8.1 Antimicrobial resistance (AMR) and emerging contaminants (ECs) removal efficiency indicators

Intense performance measurement begins with chemical measures that determine the presence of priority antibiotics and co-selective pollutants (e.g., heavy metals, disinfectants, other pharmaceuticals) in the influents and effluents, relative to the expected no effect concentrations in selecting resistance to identify hotspots and explore removal efficiency (Frascaroli et al., 2021; Hanna et al., 2023). The systematic reviews of wastewater and aquatic systems in Asia and Europe indicate that fluoroquinolones, macrolides, sulfonamides and trimethoprim tend to be abundant in WWTP effluents and receiving waters, which is why the systematic analysis of those is essential to monitor them as core EC biomarkers when evaluating performance in treatment or NbS (Frascaroli et al., 2021; Hanna et al., 2023). Meanwhile, the microbiological indicators of AMR, including culturable ARB (i.e., ESBL-producing Enterobacterales, carbapenem-resistant Gram negatives, resistant *E. coli*), are operational indicators of removal and public health relevance, and wastewater-based surveillance research indicates that improvements in clinically important ARB by treatment are measurable and, in theory, can be attributed to risk by community (Tiwari et al., 2022; Stanton et al., 2022). To monitor the environmental reservoirs, such as mangroves or agricultural catchments, integrative measures of anthropogenic AMR pressure and mitigation success are increasingly based on the indicator ARGs sulfonamide (sul1), tetracycline (tetA), and β-lactam (bla) resistance genes and mobile genetic elements (integrons, plasmids) (Lertcanawanichakul et al., 2025; Siri et al., 2023).

### 8.2 Molecular tools of antibiotic resistance genes (ARG) tracking

Performance evaluation has additionally been given central importance in the use of molecular surveillance of ARGs, where sensitive culture-independent monitoring of resistance determinants can be conducted in water, soil, sediment, and biota. Quantitative PCR (qPCR) has become a convenient standard to targeted ARG surveillance; critical reviews suggest a subset of commonly used genes, including sul1, tetA and the class 1 integron integrase intI1 as highly abundant anthropogenic targets and vanA and blaCTX M as less abundant and more clinically relevant targets together with standard protocols to sample and extract DNA, perform inhibition controls and report limits of detection to achieve comparability across studies and systems (Keenum et al., 2022). Reviews on wastewater and environmental AMR note that standardized ARG and ARB monitoring data at each treatment stage is a necessity to benchmark the effectiveness of wastewater treatment and comparisons between technologies because WWTPs can not only minimize loads but also provide an environment that allows horizontal gene transfer (La Rosa et al., 2025; Keenum et al., 2022). In addition to qPCR, metagenomics allows the complete profiling of resistomes and co occurence analysis of ARGs, mobile genetic elements and taxa; it has been employed to describe a variety of environmental reservoirs, such as mangrove sediments to Southeast Asian waters, and to identify resistance hotspots and prevailing classes of genes that are recommended to be prioritized in systems of regular qPCR assays and risk assessments (Lertcanawanichakul et al., 2025; Siri et al., 2023). Systematic maps and regional reviews emphasize that there is still heterogeneity in genes of interest targeted, units reported, and methodologies used, preventing cross-study comparisons, and thus calls on the need to harmonize molecular targets, quality control standards, and metadata standards in surveillance frameworks (Stanton et al., 2022; Keenum et al., 2022).

### 8.3 Long-term sustainability and ecosystem services assessment

The regulating performance of AMR and EC should go beyond the short-term removal metrics to long-term sustainability and preservation of ecosystem services. One Health reviews on planetary health and health reviews on One Health indicates that the presence of antibiotics and ARGs in soils, waters, and sediments and food systems are transported, and persistence and transformation are dependent on physicochemical properties, environmental conditions and land use practices, as long as there is no long term monitoring, the environmental compartments and socio economic drivers must be considered (Horvat and Kovačević, 2025; Astbury et al., 2025; Van Bavel et al., 2024). Umbrella and scoping reviews also demonstrate that food systems, primary production settings and aquatic environments are intricately bound up with AMR: heavy metals in soils and manures can be co selective to ARGs and mobile genetic elements; aquaculture and agriculture release ARB and ARGs into coastal and inland waters; natural systems including mangroves can serve as reservoirs and partial buffers of ARGs (Astbury et al., 2025; Lertcanawanichakul et al., 2025; Siri et al., 2023). The evaluation of sustainability thus means monitoring trends of antibiotic residue, ARG abundance and diversity, and co selective pollutants, whether interventions (ex, upgraded treatment, NbS restoration, reduced antibiotic usage) result in lasting reductions in environmental resistomes, and the impacts of changes on environmental ecosystem services (e.g., water purification, fisheries, food safety, and climate relevant biogeochemical cycling). It has been reviewed that sustainable and representative AMR surveillance systems need investment in data infrastructure, standardized indicators, and engagement among stakeholders, and the proper tools to do this are global surveillance platforms and standardized reporting, which allow cross-context monitoring and policy review over time (Mohammed et al., 2025). Together, these chemical, microbiological, molecular, and ecosystem-level indicators operationalize the monitoring and governance components of the microbial NbS decision framework (Figure 1). The decision framework (Figure 1) can be operationalized by aligning site-specific contaminant profiles and resistance risks with platform-specific microbial mechanisms and monitoring indicators.

Importantly, performance evaluation in microbial NbS should not be treated as a *post hoc* assessment but as an adaptive feedback mechanism informing system redesign, microbial steering, and governance decisions. Integrating chemical, microbiological, and molecular indicators into adaptive management frameworks allows NbS to be iteratively optimized to balance contaminant removal, resistance suppression, and ecosystem service preservation. Such feedback-driven monitoring operationalizes the decision framework presented in Figure 1 and enables long-term, uncertainty-aware management of AMR and EC risks.

## 9 Challenges and limitations

There are significant limitations and constraints to the scale, ecological risk, and governance of microbial technologies to nature-based or engineered systems, which limit the broader application of microbial technologies. Scalability is another constant bottleneck: in large bioreactors, conditions and yields are found not to be necessarily reproducible in the real world as it is challenging to maintain homogeneous environmental conditions, product quality, and genetic stability of strains, and capital and operating costs of infrastructure, substrates, energy, and downstream processing are high, making many microbially-derived products and remediation systems more costly than traditional ones (Birgovan et al., 2025; Vermelho et al., 2025). Local differences in soil type, climatic conditions, hydrology, pollutant mixtures, and native microbiomes imply that a microbial process that works optimally in one location could not perform in a different location but would require a long time to adapt locally, monitor itself and sometimes develop complex hybrid designs, not a plug and play solution (Banerjee et al., 2023; Wang et al., 2024). Artificially assembled microbial communities and inoculants also have to deal with competition in the resident microbes, transient resources, and spatial heterogeneity that may decrease performance with time or cause divergent community structure and functionality, making it difficult to model and to tightly control (Cheng et al., 2025; Wang et al., 2024). These technical and ecological uncertainties are associated with ecological risks and unintended consequences, especially with intervening synthetic biology-based or live microbial interventions. Synthetic biology Systematic reviews of ethics point to issues of inadvertent or intentional escape of engineered organisms, their capacity to persist, evolve, and bacterially transfer genetic material, and the impossibility of predicting the relationships of new strains with native species and ecosystems and how they might contribute to uncontrolled spread, novel food webs, or loss of biodiversity (Kurtoglu et al., 2024; Paleri and Hens, 2023). Hybrid and data driven models are under development to understand microbial community behavior better, although reviews indicate that the lack of data, unaddressed sources of bias (e.g., hyperparameter tuning, data leakage), and conceptual constraints limits their usefulness, preventing them at present to be useful to robust risk assessment and design in complex and heterogeneous settings (Cheng et al., 2025). In addition to technical and ecological problems, regulatory, ethical, and public acceptance barriers are significant limitations. Survey of microbial food and biotechnology industries points out that the regulatory processes of new microbial products, especially those with genetically modified strains or novel metabolites, can be time-consuming, disjointed across jurisdictions, and expensive in terms of safety and nutrition (Birgovan et al., 2025; Vermelho et al., 2025). Synthetic biology and microbiome engineering are the focus of ethical analyses that emphasize deontological worries of tinkering with life, biosafety and biosecurity concerns, and those of justice that have not received enough focus (Kurtoglu et al., 2024; Paleri and Hens, 2023; Hardwick et al., 2024). Studies of societal and ethical implications emphasize that people will only accept it when they view it as a demonstration of integrity, ownership, fairness, and governance; otherwise, without developers being open, engaging deeply with people, and being sensitive to other values, people will be likely to resist its implementation despite their high technical performance (Birgovan et al., 2025; Hardwick et al., 2024). There is a lack of consistent standards and easy communication about benefits, trade-offs, and monitoring plans, which affects trust and slows the market penetration of microbial or engineered foods, fears regarding invisible risks, and confusion reduction (Birgovan et al., 2025; Vermelho et al., 2025; Hardwick et al., 2024). The solutions to these issues are organized actions to enhance the scale-up and robustness of the processes, the construction of predictive ecological and risk models, and the establishment of regulatory clarity and ethical and participatory governance to ensure that microbial technologies can be utilized on a large scale without affecting the integrity of ecosystems and the social acceptability of the practice.

## 10 Integration with policy and sustainable development goals

To combine environmental AMR reduction, wastewater treatment enhancement, and resource-efficient agri-food systems with policy and the Sustainable Development Goals (SDGs), it is necessary to clearly align them with the One Health framework and the new circular bioeconomy agenda. One Health refers to an integrative and unifying strategy of achieving balance and optimization of the health of people, animals, and ecosystems through mobilization of several sectors to ensure the safety of food, water, energy, and air, combat climate change, and participation in sustainable development (Schmeyers et al., 2025). County level AMR governance reviews, AMR and food system reviews, and country level AMR governance reviews reveal that coupled human, animal and environment reservoirs cause antimicrobial use and resistance, and effective policy needs to find a way to bind the health, agriculture, environment and food safety agencies together with shared surveillance, joint risk assessment and coordinated stewardship measures, but not sectoral specific programmes (Astbury et al., 2025; Zhang Q.-Y. et al., 2025). This has a direct connection to AMR and contaminant control to SDG 3 (good health and wellbeing) by decreasing the burden of infectious diseases and enhancing health systems and SDG 6 (clean water and sanitation) by investing in WASH infrastructure and safe wastewater management, which cuts the pathogen and contaminant load (Chirgwin et al., 2021; Schmeyers et al., 2025). Sustainable agriculture and agricultural land system management is critical to SDG 2 and also to SDG 3, 6, 12, 13 and 15: systematic reviews demonstrate that well managed agriculture is a source of food security and nutrition, water use efficiency and water quality, resource efficiency, less greenhouse gas emission and less agrochemical pollution of surface waters and soils and, therefore, protects terrestrial and aquatic biodiversity as well (Viana et al., 2021). Concurrently, unsustainable intensification to achieve SDG 1 or 2 will lead to a reduction in SDG 13,14 and 15, which highlights the necessity of policies that are integrated and consider trade-offs and co-benefits in the context of goals (Viana et al., 2021). Bibliometric reviews are finding that circular economy strategies are increasingly being viewed as systemic levers toward SDG 12 (responsible consumption and production), SDG 13 (climate action), and, when conceptualized carefully, SDGs 3, 6 and 15: SDG 12 (reducing waste, resource efficiency, cutting emissions, and creating rural jobs) can, in principle, be achieved by combining circular economy strategies with bioeconomy strategies: bibliometric reviews are finding that in conceptualizations of the circular economy, SDG 12, 9 and Circular bioenergy and biohydrogen generation of organic wastes, biofertilizer use of digestates, and food waste valorization in agriculture are the example of circular bioeconomy that close nutrient and energy loops, decrease landfill application and fossil fuel reliance, and support SDG 7, 12, and 13 as well as soil quality, decreased synthetic fertilizer utilization and biodiversity protection, which are relevant to SDG 15 (Parra et al., 2025; Donkor et al., 2024). The overview of the joint application of bioeconomy and circular economy indicates that under the condition of sound sustainability standards, both approaches can help alleviate the burden on biological resources, preserve biodiversity and reinforce the primary food production role of agriculture, although it is also stated that, in the absence of appropriate governance, the growth of the bioeconomy may aggravate competition in land use, endangering food access, and hence, in this case, strong safeguards and equity-focused policy formulations are necessary (Ferraz and Pyka, 2023; Abad-Segura et al., 2021). Circular economy, Industry 4.0 and SDGs policy oriented reviews also note that the digital and process innovations (e.g., remote sensing, data platforms, automation) have the potential to facilitate more resource efficient and low-emission production systems, but point out that a gap in explicit policy focus on the topic persists: the governance structures and indicators through which circular bioeconomy projects are linked to SDGs and the One Health outcomes are underdeveloped and dispersed (Ferraz and Pyka, 2023; Baca-Neglia et al., 2025). In general, AMR and contaminant control, WASH, circular bioeconomy, and sustainable agriculture must be aligned with One Health and SDGs require context-sensitive, integrated policy, which coordinates many sectors, balances trade-offs, and tracks outcomes in the form of indicators tailored to the context and time.

## 11 Future perspectives and research gaps

Building on the challenges outlined in Section 9, emerging studies into microbial nature-based solutions (NbS) should directly contemplate how climate change will transform microbial communities and functions, how digital technologies can be used to design and control, and overcome the long-standing disconnect between laboratory success and field dependability. Temperature, moisture regimes and disturbance patterns in soils and waters are already being changed by climatic change, and have a strong implication on microbial community composition, decomposition, nutrient cycling and plant-soil feedbacks, which form the basis of many NbS, yet this change demonstrably alters responses and impacts on predicting ecosystem resilience; longer-term syntheses have revealed that bacteria, fungi and archaea, as a collective, regulate plant-soil ecosystems and ecosystem resilience, but also indicate that responses and impacts are highly context-dependent and poorly captured in predictive models (Wang et al., 2024; Corredor et al., 2024). Recent reviews on NbS and climate resilience suggest that significant gaps in knowledge persist regarding the performance or operation of the various types of NbS under changing climate conditions, their scalability and transferability across a broader range of geographic areas, and their long-term impact on ecosystem services and community wellbeing, which is why there is a pressing need to conduct empirical experiments of multiple years that integrate microbial, biogeochemical, and social data across a spectrum of changing climatic conditions (Alikhanova and Bull, 2023; Vazin et al., 2024).

Across these emerging directions, three priority research frontiers can be identified: (i) long-term, field-scale validation of microbial NbS under climate variability and mixed contaminant loads; (ii) integration of resistome dynamics and horizontal gene transfer metrics into NbS design and performance benchmarks; and (iii) development of governance-ready indicators that link microbial processes, ecosystem services, and public health outcomes. Addressing these priorities is critical for translating laboratory-scale innovations into reliable, scalable, and policy-relevant interventions.

Simultaneously, the fast development of artificial intelligence (AI), machine learning (ML), Internet of Things (IoT) and AIoT in the field of agriculture and environmental management presents potent means to track, model, and optimize microbial NbS. Agriculture Systematic reviews underscore that incorporating ML into heterogeneous data streams that can include satellite/drone images, IoT sensor networks, and weather data can help define the intricate agroecosystems and assist in managing them in an adaptive and resource efficient way, although obstacles exist regarding data quality, inter-site transferability, and human-machine interactions (Araújo et al., 2023; Adli et al., 2023). Similar studies in bioremediation suggest that the world needs standardized, global databases on soil and microbiomes and neural network bioclimatic models to predict human development and environmental engineering through AI-aided mapping, early warning, and engineered microbial interventions to promote human development and environmental health (Corredor et al., 2024). Nevertheless, in general, the state of AI/ML applications today is marked by the lack of data, a lack of validations in the real world, and ethical and governance issues, which indicates that the next generation of digital twins of microbial NbS needs open data infrastructures, participatory design, and the explicit consideration of uncertainty and bias (Araújo et al., 2023; Corredor et al., 2024). One of the largest and most frequent research gaps follows the translation of laboratory-scale microbial innovations, including glyphosate-degrading bacteria, PAH degrading consortia, or photosynthetic microbe-based nanomaterials into robust field-scale processes. Reviews of microbial bioremediation and pollutant degradation have found that promising strains and pathways often fail in their performance after being subjected to changing field conditions, mixed contaminants, and interaction with native microbiota, and large-scale deployment is costly due to economic and logistical factors (Souza et al., 2025; Banerjee et al., 2023). Surveys of microbial technologies to develop sustainable food systems also note the high cost of scaling, regulatory barriers, and social acceptability, and modular and flexible biotechnology platforms that can respond to climate change and market shocks as an impediment to their use (Birgovan et al., 2025). The only way of closing this divide will be through multi-site pilot projects, which will explicitly address scalability, long-term observations of ecological and socio-economic measures, and more explicit integration of omics, process engineering, and AI-controlled control systems. In general, future directions are convergent and integrate the necessity of interdisciplinary programs that pair climate-conscious experimentation, digital and AI optimization of the system, implementation science that is concerned with real-world and field-scale microbial NbS, resilience-based, equitable, and compatible with the transition to sustainability.

## 12 Conclusions

Microbial innovations position nature-based solutions (NbS) as promising and sustainable approaches to the increasing problems of antimicrobial resistance (AMR) and emerging contaminants (ECs). Together, Figures 1–3 provide a coherent progression from evidence synthesis (Figure 2) to mechanistic understanding at the plant–microbe and microbial community level (Figure 3), culminating in a decision-ready framework for designing and managing microbial-driven nature-based solutions for AMR and EC mitigation (Figure 1). The decision framework (Figure 1) can be operationalized by aligning site-specific contaminant profiles and resistance risks with platform-specific microbial mechanisms and monitoring indicators. The key insights of this review are as follows: microbial diversity, functional consortia, and interactions of microbes and plants play a central role in degrading, transforming, and immobilizing antibiotics, resistance genes, and complex pollutants in soil and aquatic environments. The importance of microbial innovations to NbS is that they can be used to increase the efficiency of remediation without compromising ecosystem integrity, decrease the amount of chemicals applied, and minimize the selective pressure toward the development of resistance. Novel technologies, including omics technologies, microbial consortia engineering, and bioelectrochemical systems, are also enhancing the functionality and stability of NbS platforms. In the future, sustainable mitigation of AMR and ECs will involve the combination of microbial NbS with the One Health paradigm, enhanced long-term monitoring, biosafety, and scaling of site-specific solutions with the aid of supportive policies and interdisciplinary research. This review demonstrates that nature-based solutions are not inherently protective against antimicrobial resistance but require deliberate microbial and system-level design to avoid unintended resistance amplification. By integrating microbial mechanisms, NbS platform trade-offs, monitoring strategies, and governance considerations within a unified decision framework, this work advances NbS from descriptive concepts toward decision-ready interventions. The synthesis presented here provides a foundation for designing scalable, biosafe, and policy-relevant microbial NbS capable of mitigating antimicrobial resistance and emerging contaminants within a One Health and sustainability context.

## Data Availability

The raw data supporting the conclusions of this article will be made available by the authors, without undue reservation.

## References

[B1] Abad-Segura E. Batlles-delaFuente A. González-Zamar M.-D. Belmonte-Ureña L. J. (2021). Implications for sustainability of the joint application of bioeconomy and circular economy: a worldwide trend study. Sustainability 13:7182. doi: 10.3390/su13137182

[B2] Abou-Kandil A. Shibli A. Azaizeh H. Wolff D. Wick A. Jadoun J. (2020). Fate and removal of bacteria and antibiotic resistance genes in horizontal subsurface constructed wetlands: effect of mixed vegetation and substrate type. Sci. Total Environ. 759:144193. doi: 10.1016/j.scitotenv.2020.144193 33338689

[B3] Adli H. K. Remli M. A. Wong K. N. S. W. S. Ismail N. A. González-Briones A. Corchado J. M. . (2023). Recent advancements and challenges of AIoT application in smart agriculture: a review. Sensors 23:3752. doi: 10.3390/s23073752 37050812 PMC10098529

[B4] Ahmad H. A. Ahmad S. Cui Q. Wang Z. Wei H. Chen X. . (2021). The environmental distribution and removal of emerging pollutants, highlighting the importance of using microbes as a potential degrader: a review. Sci. Total Environ. 809:151926. doi: 10.1016/j.scitotenv.2021.151926 34838908

[B5] Ajao V. Nam K. Chatzopoulos P. Spruijt E. Bruning H. Rijnaarts H. . (2020). Regeneration and reuse of microbial extracellular polymers immobilised on a bed column for heavy metal recovery. Water Res. 171:115472. doi: 10.1016/j.watres.2020.115472 31931379

[B6] Ajaz S. Hassan A. A. Michael R. N. Leusch F. D. L. (2024). Removal of organic micropollutants in biologically active filters: a systematic quantitative review of key influencing factors. J. Environ. Manage. 353:120203. doi: 10.1016/j.jenvman.2024.120203 38325285

[B7] Alexandrino D. A. Mucha A. P. Almeida C. M. R. Gao W. Jia Z. Carvalho M. F. (2017). Biodegradation of the veterinary antibiotics enrofloxacin and ceftiofur and associated microbial community dynamics. Sci. Total Environ. 581–582, 359–368. doi: 10.1016/j.scitotenv.2016.12.141 28069302

[B8] Alikhanova S. Bull J. W. (2023). Review of nature-based solutions in dryland ecosystems: the aral sea case study. Environ. Manage. 72, 457–472. doi: 10.1007/s00267-023-01822-z 37115238 PMC10372098

[B9] Almenar J. B. Elliot T. Rugani B. Philippe B. Gutierrez T. N. Sonnemann G. . (2020). Nexus between nature-based solutions, ecosystem services and urban challenges. Land Use Policy 100:104898. doi: 10.1016/j.landusepol.2020.104898

[B10] Anedda E. Farrell M. L. Morris D. Burgess C. M. (2023). Evaluating the impact of heavy metals on antimicrobial resistance in the primary food production environment: a scoping review. Environ. Pollut. 320:121035. doi: 10.1016/j.envpol.2023.121035 36623784

[B11] Araújo S. O. Peres R. S. Ramalho J. C. Lidon F. Barata J. (2023). Machine learning applications in agriculture: current trends, challenges, and future perspectives. Agronomy 13:2976. doi: 10.3390/agronomy13122976

[B12] Arias-Castro E. Castrejón-Godínez M. L. Mussali-Galante P. Tovar-Sánchez E. Rodríguez A. (2025). Pesticides degradation through microorganisms immobilized on agro-industrial waste: a promising approach for their elimination from aquatic environments. Processes 13:1073. doi: 10.3390/pr13041073

[B13] Aslam M. B. Rehman T. U. R. Alavi A. F. Haleem A. Haq A. Ahmed S. . (2025). Design, construction and efficiency analysis of bagasse-based charcoal packed horizontal lab-scale wetland for the removal of antibiotic-resistant bacteria. BMC Microbiol. 25:418. doi: 10.1186/s12866-025-04061-w 40618018 PMC12228385

[B14] Astbury C. C. Hu C. Sivapragasam K. Rajan A. Geise M. Aenishaenslin C. . (2025). Interconnections between the food system and antimicrobial resistance: a systems-informed umbrella review from a One Health perspective. One *Health* 21:101143. doi: 10.1016/j.onehlt.2025.101143 PMC12311529 40746419

[B15] Baca-Neglia M. Barreto-Pio C. Virú-Vásquez P. Badillo-Rivera E. Césare-Coral M. F. Castro-Pantoja J. B. . (2025). Industry 4.0, circular economy and sustainable development goals: future research directions through scientometrics and mini-review. Sustainability 17:6468. doi: 10.3390/su17146468

[B16] Banerjee S. Gupta N. Pramanik K. Gope M. GhoshThakur R. Karmakar A. . (2023). Microbes and microbial strategies in carcinogenic polycyclic aromatic hydrocarbons remediation: a systematic review. Environ. Sci. Pollut. Res. 31, 1811–1840. doi: 10.1007/s11356-023-31140-0 38063960

[B17] Barancheshme F. Munir M. (2018). Strategies to combat antibiotic resistance in the wastewater treatment plants. Front. Microbiol. 8:2603. doi: 10.3389/fmicb.2017.02603 29387043 PMC5776126

[B18] Bertrans-Tubau L. Martínez-Campos S. Lopez-Doval J. Abril M. Ponsá S. Salvadó V. . (2024). Nature-based bioreactors: Tackling antibiotic resistance in urban wastewater treatment. Environ. Sci. Ecotechnol. 22:100445. doi: 10.1016/j.ese.2024.100445 39055482 PMC11269294

[B19] Birgovan A. L. Lakatos E. S. Cioca L. I. Paul N. L. Vatca S. D. Kis E. . (2025). Harnessing microbial power for a sustainable future food system. Microorganisms 13:2217. doi: 10.3390/microorganisms13092217 41011548 PMC12472289

[B20] Brienza M. Sauvêtre A. Ait-Mouheb N. Bru-Adan V. Coviello D. Lequette K. . (2022). Reclaimed wastewater reuse in irrigation: role of biofilms in the fate of antibiotics and spread of antimicrobial resistance. Water Res. 221:118830. doi: 10.1016/j.watres.2022.118830 35841791

[B21] Chen J. Ying G.-G. Wei X.-D. Liu Y.-S. Liu S.-S. Hu L.-X. . (2016). Removal of antibiotics and antibiotic resistance genes from domestic sewage by constructed wetlands: effect of flow configuration and plant species. Sci. Total Environ. 571, 974–982. doi: 10.1016/j.scitotenv.2016.07.085 27443461

[B22] Cheng Z. Xia W. Zhu J.-J. Cao J. Ren Z. J. Yuan H. (2025). A comprehensive guideline for hybrid modeling of engineered microbial processes. Water Res. 288(Pt A):124559. doi: 10.1016/j.watres.2025.124559 40929841

[B23] Cheung C. Naughton P. J. Dooley J. S. G. Corcionivoschi N. Brooks C. (2025). The spread of antimicrobial resistance in the aquatic environment from faecal pollution: a scoping review of a multifaceted issue. Environ. Monit. Assess. 197:467. doi: 10.1007/s10661-025-13860-7 40131552 PMC11937110

[B24] Chirgwin H. Cairncross S. Zehra D. Waddington H. S. (2021). Interventions promoting uptake of water, sanitation and hygiene (WASH) technologies in low- and middle-income countries: an evidence and gap map of effectiveness studies. Campbell Syst. Rev. 17:e1194. doi: 10.1002/cl2.1194 36951806 PMC8988822

[B25] Corredor D. Duchicela J. Flores F. J. Maya M. Guerron E. (2024). Review of explosive contamination and bioremediation: insights from microbial and bio-omic approaches. Toxics 12:249. doi: 10.3390/toxics12040249 38668472 PMC11053648

[B26] Cui E. Cui B. Fan X. Li S. Gao F. (2021). Ryegrass (*Lolium multiflorum* L.) and Indian mustard (*Brassica juncea* L.) intercropping can improve the phytoremediation of antibiotics and antibiotic resistance genes but not heavy metals. Sci. Total Environ. 784:147093. doi: 10.1016/j.scitotenv.2021.147093 33895506

[B27] Cusumano G. Flores G. A. Venanzoni R. Angelini P. Zengin G. (2025). Green solutions to a growing problem: harnessing plants for antibiotic removal from the environment. Antibiotics 14:1031. doi: 10.3390/antibiotics14101031 41148723 PMC12562217

[B28] Da Silva V. F. Wolff D. B. (2024). Removal of antibiotics in constructed wetlands: a review and bibliometric analysis. Anais Da Academia Brasileira De Ciências 96:e20240275. doi: 10.1590/0001-3765202420240275 39570174

[B29] Das N. Pandey P. (2025). Biochar-driven rhizoremediation of soil contaminated with organic pollutants: engineered solutions, microbiome enrichment, and bioeconomic benefits for ecosystem restoration. Biochar 7:101. doi: 10.1007/s42773-025-00491-x

[B30] Davis B. C. Brown C. Gupta S. Calarco J. Liguori K. Milligan E. . (2023). Recommendations for the use of metagenomics for routine monitoring of antibiotic resistance in wastewater and impacted aquatic environments. Crit. Rev. Environ. Sci. Technol. 53, 1731–1756. doi: 10.1080/10643389.2023.2181620

[B31] Deza-Cruz I. De Menezes A. Gardner B. Aktan Í. Alnajjar S. Betson M. . (2025). Mapping the evidence of the effects of environmental factors on the prevalence of antibiotic resistance in the non-built environment. Environ. Int. 202:109634. doi: 10.1016/j.envint.2025.109634 40753756

[B32] Di Carlo P. Serra N. Alduina R. Guarino R. Craxì A. Giammanco A. . (2022). A systematic review on omics data (metagenomics, metatranscriptomics, and metabolomics) in the role of microbiome in gallbladder disease. Front. Physiol. 13:888233. doi: 10.3389/fphys.2022.888233 36111147 PMC9468903

[B33] Do P. C. Assefa Y. A. Batikawai S. M. Reid S. A. (2023). Strengthening antimicrobial resistance surveillance systems: a scoping review. BMC Infect. Dis. 23:593. doi: 10.1186/s12879-023-08585-2 37697310 PMC10496311

[B34] Donkor E. S. Odoom A. Osman A.-H. Darkwah S. Kotey F. C. N. (2024). A systematic review on antimicrobial resistance in ghana from a One Health perspective. Antibiotics 13:662. doi: 10.3390/antibiotics13070662 39061344 PMC11274323

[B35] Egbuna C. Amadi C. N. Patrick-Iwuanyanwu K. C. Ezzat S. M. Awuchi C. G. Ugonwa P. O. . (2021). Emerging pollutants in Nigeria: a systematic review. Environ. Toxicol. Pharmacol. 85:103638. doi: 10.1016/j.etap.2021.103638 33757839

[B36] Eke S. M. Cua A. (2025). Invisible engines of resistance: how global inequities drive antimicrobial failure. Antibiotics 14:659. doi: 10.3390/antibiotics14070659 40723962 PMC12291774

[B37] Elsayad R. M. Sharshir S. W. Khalil A. Basha A. M. (2024). Recent advancements in wastewater treatment via anaerobic fermentation process: a systematic review. J. Environ. Manage. 366:121724. doi: 10.1016/j.jenvman.2024.121724 38971071

[B38] Enshaie E. Nigam S. Patel S. Rai V. (2025). Livestock antibiotics use and antimicrobial resistance. Antibiotics 14:621. doi: 10.3390/antibiotics14060621 40558211 PMC12189104

[B39] Esraz-Ul-Zannat M. d. Dedekorkut-Howes A. Morgan E. A. (2024). A review of nature-based infrastructures and their effectiveness for urban flood risk mitigation. Wiley Interdiscipl. Rev. Clim. Change 15:e889. doi: 10.1002/wcc.889

[B40] Fang X. Li J. Ma Q. (2023). Integrating green infrastructure, ecosystem services and nature-based solutions for urban sustainability: a comprehensive literature review. Sustain. Cities Soc. 98:104843. doi: 10.1016/j.scs.2023.104843

[B41] Ferraz D. Pyka A. (2023). Circular economy, bioeconomy, and sustainable development goals: a systematic literature review. Environ. Sci. Pollut. Res. doi: 10.1007/s11356-023-29632-0. [Epub ahead of print]. 37702868

[B42] Frascaroli G. Reid D. Hunter C. Roberts J. Helwig K. Spencer J. . (2021). Pharmaceuticals in wastewater treatment plants: a systematic review on the substances of greatest concern responsible for the development of antimicrobial resistance. Appl. Sci. 11:6670. doi: 10.20944/preprints202106.0672.v1

[B43] García J. García-Galán M. J. Day J. W. Boopathy R. White J. R. Wallace S. . (2020). A review of emerging organic contaminants (EOCs), antibiotic resistant bacteria (ARB), and antibiotic resistance genes (ARGs) in the environment: increasing removal with wetlands and reducing environmental impacts. Bioresour. Technol. 307:123228. doi: 10.1016/j.biortech.2020.123228 32247686

[B44] Gentile A. Piccolo P. Iannece P. Cicatelli A. Castiglione S. Guarino F. (2024). Reduction of antimicrobial resistance: advancements in nature-based wastewater treatment. J. Hazard. Mater. 471:134330. doi: 10.1016/j.jhazmat.2024.134330 38678704

[B45] Gong W. Guo L. Huang C. Xie B. Jiang M. Zhao Y. . (2024). A systematic review of antibiotics and antibiotic resistance genes (ARGs) in mariculture wastewater: antibiotics removal by microalgal-bacterial symbiotic system (MBSS), ARGs characterization on the metagenomic. Sci. Total Environ. 930:172601. doi: 10.1016/j.scitotenv.2024.172601 38657817

[B46] Hamers R. L. Dobreva Z. Cassini A. Tamara A. Lazarus G. Asadinia K. S. . (2023). Global knowledge gaps on antimicrobial resistance in the human health sector: a scoping review. Int. J. Infect. Dis. 134, 142–149. doi: 10.1016/j.ijid.2023.06.004 37301361

[B47] Hanna N. Tamhankar A. J. Lundborg C. S. (2023). Antibiotic concentrations and antibiotic resistance in aquatic environments of the WHO Western Pacific and South-East Asia regions: a systematic review and probabilistic environmental hazard assessment. Lancet Planetary Health 7, e45–e54. doi: 10.1016/S2542-5196(22)00254-6 36608948

[B48] Hardwick A. Cummings C. Graves J. Kuzma J. (2024). Can societal and ethical implications of precision microbiome engineering be applied to the built environment? A systematic review of the literature. Environ. Syst. Decisions 44, 215–238. doi: 10.1007/s10669-024-09965-y

[B49] Hazra M. Joshi H. Williams J. B. Watts J. E. (2022). Antibiotics and antibiotic resistant bacteria/genes in urban wastewater: a comparison of their fate in conventional treatment systems and constructed wetlands. Chemosphere 303(Pt 2):135148. doi: 10.1016/j.chemosphere.2022.135148 35640694

[B50] Hnini M. Rabeh K. Oubohssaine M. (2024). Interactions between beneficial soil microorganisms (PGPR and AMF) and host plants for environmental restoration: a systematic review. Plant Stress 11:100391. doi: 10.1016/j.stress.2024.100391

[B51] Horvat O. Kovačević Z. (2025). Human and veterinary medicine collaboration: synergistic approach to address antimicrobial resistance through the lens of planetary health. Antibiotics 14:38. doi: 10.3390/antibiotics14010038 39858324 PMC11762137

[B52] Jagaba A. H. Kutty S. R. M. Noor A. Birniwa A. H. Affam A. C. Lawal I. M. . (2021). A systematic literature review of biocarriers: central elements for biofilm formation, organic and nutrients removal in sequencing batch biofilm reactor. J. Water Process Eng. 42:102178. doi: 10.1016/j.jwpe.2021.102178

[B53] Keenum I. Liguori K. Calarco J. Davis B. C. Milligan E. Harwood V. J. . (2022). A framework for standardized qPCR-targets and protocols for quantifying antibiotic resistance in surface water, recycled water and wastewater. Crit. Rev. Environ. Sci. Technol. 52, 4395–4419. doi: 10.1080/10643389.2021.2024739

[B54] Kiruba N J. M. Saeid A. (2022). An insight into microbial inoculants for bioconversion of waste biomass into sustainable “bio-organic” fertilizers: a bibliometric analysis and systematic literature review. Int. J. Mol. Sci. 23:13049. doi: 10.3390/ijms232113049 36361844 PMC9656562

[B55] Kotoky R. Rajkumari J. Pandey P. (2018). The rhizosphere microbiome: Significance in rhizoremediation of polyaromatic hydrocarbon contaminated soil. J. Environ. Manage. 217, 858–870. doi: 10.1016/j.jenvman.2018.04.022 29660711

[B56] Kotrbová L. Grabicová K. Švecová H. Stanová A. V. Petrlíková M. Grabic R. . (2025). The effect of WWTP products amendments on Phaseolus vulgaris rhizosphere and its ability to inactivate clarithromycin. Sci. Rep. 15:30950. doi: 10.1038/s41598-025-14953-6 40846880 PMC12373788

[B57] Kurtoglu A. Yildiz A. Arda B. (2024). The view of synthetic biology in the field of ethics: a thematic systematic review. Front. Bioeng. Biotechnol. 12:1397796. doi: 10.3389/fbioe.2024.1397796 38863492 PMC11165145

[B58] Kuzyanov D. Panchenko L. Pozdnyakova N. Muratova A. (2025). *Medicago sativa* L. root exudation of phenolic compounds and effect of flavonoids on phenanthrene degradation by two rhizobacteria. Front. Bioscience-Elite 17:25779. doi: 10.31083/FBE25779 40150983

[B59] La Rosa M. C. Maugeri A. Favara G. La Mastra C. Lio R. M. S. Barchitta M. . (2025). The impact of wastewater on antimicrobial resistance: a scoping review of transmission pathways and contributing factors. Antibiotics 14:131. doi: 10.3390/antibiotics14020131 40001375 PMC11851908

[B60] Lasch C. Myronovskyi M. Luzhetskyy A. (2025). Streptomyces as a versatile host platform for heterologous production of microbial natural products. Nat. Product Rep. 43, 371–390. doi: 10.1039/d5np00036j 40923873

[B61] Le T.-H. Ng C. Tran N. H. Chen H. Gin K. Y.-H. (2018). Removal of antibiotic residues, antibiotic resistant bacteria and antibiotic resistance genes in municipal wastewater by membrane bioreactor systems. Water Res. 145, 498–508. doi: 10.1016/j.watres.2018.08.060 30193193

[B62] Lertcanawanichakul M. Bhoopong P. Horpet P. (2025). Mangrove ecosystems as reservoirs of antibiotic resistance genes: a narrative review. Antibiotics 14:1022. doi: 10.3390/antibiotics14101022 41148714 PMC12561704

[B63] Li J. Chen L. Jin S. Huang L. Chen H. (2025). Influences of plants and soil microbes on antibiotics in the rhizosphere: a review. Plant Soil Environ. 71, 67–92. doi: 10.17221/350/2024-PSE

[B64] Liu X. Guo X. Liu Y. Lu S. Xi B. Zhang J. . (2019). A review on removing antibiotics and antibiotic resistance genes from wastewater by constructed wetlands: Performance and microbial response. Environ. Pollut. 254(Pt A):112996. doi: 10.1016/j.envpol.2019.112996 31400665

[B65] Luo H. Liu C. He D. Xu J. Sun J. Li J. . (2021). Environmental behaviors of microplastics in aquatic systems: a systematic review on degradation, adsorption, toxicity and biofilm under aging conditions. J. Hazardous Mater. 423(Pt A):126915. doi: 10.1016/j.jhazmat.2021.126915 34461541

[B66] Lv M. Zhang D. Niu X. Ma J. Lin Z. Fu M. (2022). Insights into the fate of antibiotics in constructed wetland systems: Removal performance and mechanisms. J. Environ. Manage. 321:116028. doi: 10.1016/j.jenvman.2022.116028 36104874

[B67] Mahmud F. A. Islam M. A. Rubel M. H. Mukharjee S. K. Kumar M. Bhattacharya P. . (2023). Effects of halotolerant rhizobacteria on rice seedlings under salinity stress. Sci. Total Environ. 892:163774. doi: 10.1016/j.scitotenv.2023.163774 37230352

[B68] Mahto K. U. Vandana Priyadarshanee M. Samantaray D. P. Das S. (2022). Bacterial biofilm and extracellular polymeric substances in the treatment of environmental pollutants: Beyond the protective role in survivability. J. Clean. Prod. 379:134759. doi: 10.1016/j.jclepro.2022.134759

[B69] Maldonado M. P. Ofori-Darko D. Nichols V. French J. Spence K. Reid-Smith R. J. . (2025). Investigating the occurrence of antimicrobial resistance in the environment in Canada: a scoping review. Can. J. Microbiol. 71, 1–13. doi: 10.1139/cjm-2024-0189 40279669

[B70] Manzoor M. Guan D.-X. Q L. M. A. (2025). Plant-microbiome interactions for enhanced crop production under cadmium stress: a review. Sci. Total Environ. 965:178538. doi: 10.1016/j.scitotenv.2025.178538 39879949

[B71] Mao Y. Zhao Y. Cotterill S. (2023). Examining current and future applications of electrocoagulation in wastewater treatment. Water 15:1455. doi: 10.3390/w15081455

[B72] Marín O. González B. Poupin M. J. (2021). From microbial dynamics to functionality in the rhizosphere: a systematic review of the opportunities with synthetic microbial communities. Front. Plant Sci. 12:650609. doi: 10.3389/fpls.2021.650609 34149752 PMC8210828

[B73] Marzouk S. H. Kwaslema D. R. Omar M. M. Mohamed S. H. (2024). “Harnessing the power of soil microbes: their dual impact in integrated nutrient management and mediating climate stress for sustainable rice crop production”: a systematic review. Heliyon 11:e41158. doi: 10.2139/ssrn.4763320 39758363 PMC11699367

[B74] McCorquodale-Bauer K. Grosshans R. Zvomuya F. Cicek N. (2023). Critical review of phytoremediation for the removal of antibiotics and antibiotic resistance genes in wastewater. Sci. Total Environ. 870:161876. doi: 10.1016/j.scitotenv.2023.161876 36716878

[B75] Mishra A. Singh L. Singh D. (2022). Unboxing the black box—one step forward to understand the soil microbiome: a systematic review. Microb. Ecol. 85, 669–683. doi: 10.1007/s00248-022-01962-5 35112151 PMC9957845

[B76] Mishra S. Cheng L. Lian Y. (2023). Response of biofilm–based systems for antibiotics removal from wastewater: resource efficiency and process resiliency. Chemosphere 340:139878. doi: 10.1016/j.chemosphere.2023.139878 37604340

[B77] Mohammed E. A. H. Kovács B. Kuunya R. Mustafa E. O. A. Abbo A. S. H. Pál K. (2025). Antibiotic resistance in aquaculture: challenges, trends analysis, and alternative approaches. Antibiotics 14:598. doi: 10.3390/antibiotics14060598 40558188 PMC12189707

[B78] Mu X. Huang Z. Ohore O. E. Yang J. Peng K. Li S. . (2023). Impact of antibiotics on microbial community in aquatic environment and biodegradation mechanism: a review and bibliometric analysis. Environ. Sci. Pollut. Res. 30, 66431–66444. doi: 10.1007/s11356-023-27018-w 37101213

[B79] Nasrollahi N. Vatanpour V. Khataee A. (2022). Removal of antibiotics from wastewaters by membrane technology: limitations, successes, and future improvements. Sci. Total Environ. 838(Pt 1):156010. doi: 10.1016/j.scitotenv.2022.156010 35595150

[B80] Nazir A. Nazir A. Nazir A. Nazir A. Zuhair V. Aman S. . (2025). The global challenge of antimicrobial resistance: mechanisms, case studies, and mitigation approaches. Health Sci. Rep. 8:e71077. doi: 10.1002/hsr2.71077 40704322 PMC12284435

[B81] Orr J. A. Armitage D. W. Letten A. D. (2025). Coexistence theory for microbial ecology, and vice versa. Environ. Microbiol. 27:e70072. doi: 10.1111/1462-2920.70072 40033656 PMC11876725

[B82] Pagliaccia B. Carretti E. Severi M. Berti D. Lubello C. Lotti T. (2021). Heavy metal biosorption by extracellular polymeric substances (EPS) recovered from anammox granular sludge. J. Hazardous Mater. 424(Pt C):126661. doi: 10.1016/j.jhazmat.2021.126661 34315635

[B83] Paleri V. A. Hens K. (2023). Beyond the organism versus machine dichotomy: a review of ethical concerns in synthetic biology. ACS Synth. Biol. 13, 3–14. doi: 10.1021/acssynbio.3c00456 38070167

[B84] Parra R. Chicaiza-Ortiz C. Herrera-Feijoo R. J. Arellano-Yasaca D. V. Lee L.-C. Supe-Tulcan R. X. . (2025). Advancements of biohydrogen production based on anaerobic digestion: technologies, substrates, and future prospects. Science 7:52. doi: 10.3390/sci7020052

[B85] Pastor-Lopez E. J. Casas M. E. Hellman D. Müller J. A. Matamoros V. (2024). Nature-based solutions for antibiotics and antimicrobial resistance removal in tertiary wastewater treatment: microbiological composition and risk assessment. Water Res. 261:122038. doi: 10.1016/j.watres.2024.122038 38996727

[B86] Paul I. Das R. Halder G. (2025). Decoding the interactions between antibiotics and microplastics-chemistry, environmental impacts, and mitigation approaches- a state-of-the-art review. Environ. Res. 286(Pt 1):122774. doi: 10.1016/j.envres.2025.122774 40915478

[B87] Perković S. Paul C. Vasić F. Helming K. (2022). Human health and soil health risks from heavy metals, micro(nano)plastics, and antibiotic resistant bacteria in agricultural soils. Agronomy 12:2945. doi: 10.3390/agronomy12122945

[B88] Priyadarshanee M. Das S. (2024). Spectra metrology for interaction of heavy metals with extracellular polymeric substances (EPS) of *Pseudomonas aeruginosa* OMCS-1 reveals static quenching and complexation dynamics of EPS with heavy metals. J. Hazard. Mater. 466:133617. doi: 10.1016/j.jhazmat.2024.133617 38306836

[B89] Qiu D. Ke M. Zhang Q. Zhang F. Lu T. Sun L. . (2022). Response of microbial antibiotic resistance to pesticides: an emerging health threat. Sci. Total Environ. 850:158057. doi: 10.1016/j.scitotenv.2022.158057 35977623

[B90] Sabri N. A. Schmitt H. Van Der Zaan B. M. Gerritsen H. W. Rijnaarts H. H. M. Langenhoff A. A. M. (2021). Performance of full scale constructed wetlands in removing antibiotics and antibiotic resistance genes. Sci. Total Environ. 786:147368. doi: 10.1016/j.scitotenv.2021.147368 33965831

[B91] Saidulu D. Majumder A. Gupta A. K. (2021). A systematic review of moving bed biofilm reactor, membrane bioreactor, and moving bed membrane bioreactor for wastewater treatment: comparison of research trends, removal mechanisms, and performance. J. Environ. Chem. Eng. 9:106112. doi: 10.1016/j.jece.2021.106112

[B92] Schmeyers L. Thomschke S. Mende L. V. Stichel G. Schiller D. Fleßa S. (2025). Economic methods and spatial scales in One Health: results from a scoping review. One *Health* 21:101115. doi: 10.1016/j.onehlt.2025.101115 PMC12242469 40641675

[B93] Shayista H. Prasad M. N. N. Raj S. N. Prasad A. Lakshmi S. Ranjini H. K. . (2024). Complexity of antibiotic resistance and its impact on gut microbiota dynamics. Eng. Microbiol. 5:100187. doi: 10.1016/j.engmic.2024.100187 40538717 PMC12173824

[B94] Sherwani M. K. Ruuskanen M. O. Feldner-Busztin D. Firbas P. N. Boza G. Móréh Á. . (2025). Multi-omics time-series analysis in microbiome research: a systematic review. Brief. Bioinformatics 26, 1–27. doi: 10.1093/bib/bbaf502 PMC12499790 41052276

[B95] Siri Y. Precha N. Sirikanchana K. Haramoto E. Makkaew P. (2023). Antimicrobial resistance in southeast Asian water environments: a systematic review of current evidence and future research directions. Sci. Total Environ. 896:165229. doi: 10.1016/j.scitotenv.2023.165229 37394072

[B96] Souza K. S. Da Silva M. R. F. Candido M. A. Lins H. T. S. De Lima Torres G. Da Silva Felix K. C. . (2025). Biodegradation potential of glyphosate by bacteria: a systematic review on metabolic mechanisms and application strategies. Agronomy 15:1247. doi: 10.3390/agronomy15051247

[B97] Stanton I. C. Bethel A. Leonard A. F. C. Gaze W. H. Garside R. (2022). Existing evidence on antibiotic resistance exposure and transmission to humans from the environment: a systematic map. Environ. Evid. 11:8. doi: 10.1186/s13750-022-00262-2 35308196 PMC8917330

[B98] Sun X.-L. Xiang H. Xiong H.-Q. Fang Y.-C. Wang Y. (2022). Bioremediation of microplastics in freshwater environments: a systematic review of biofilm culture, degradation mechanisms, and analytical methods. Sci. Total Environ. 863:160953. doi: 10.1016/j.scitotenv.2022.160953 36543072

[B99] Tiwari A. Kurittu P. Al-Mustapha A. I. Heljanko V. Johansson V. Thakali O. . (2022). Wastewater surveillance of antibiotic-resistant bacterial pathogens: a systematic review. Front. Microbiol. 13:977106. doi: 10.3389/fmicb.2022.977106 36590429 PMC9798455

[B100] Uruén C. Chopo-Escuin G. Tommassen J. Mainar-Jaime R. C. Arenas J. (2020). Biofilms as promoters of bacterial antibiotic resistance and tolerance. Antibiotics 10:3. doi: 10.3390/antibiotics10010003 33374551 PMC7822488

[B101] Uy M. J. Robles M. E. Oh Y. Haque M. T. Mueca C. C. Kim L.-H. (2025). Biodiversity monitoring in constructed wetlands: a systematic review of assessment methods and ecosystem functions. Diversity 17:367. doi: 10.3390/d17050367

[B102] Van Bavel B. Berrang-Ford L. Moon K. Gudda F. Thornton A. J. Robinson R. F. S. . (2024). Intersections between climate change and antimicrobial resistance: a systematic scoping review. Lancet Planetary Health 8, e1118–e1128. doi: 10.1016/S2542-5196(24)00273-0 39674199

[B103] Vandana Priyadarshanee M. Das S. (2023). Bacterial extracellular polymeric substances: biosynthesis and interaction with environmental pollutants. Chemosphere 332:138876. doi: 10.1016/j.chemosphere.2023.138876 37164199

[B104] Vasarevičius S. Paliuliene V. (2025). Immobilization of cadmium, lead, and copper in soil using bacteria: a literature review. Land 14:1547. doi: 10.3390/land14081547

[B105] Vazin F. Chan D. W. M. Hanaee T. Sarvari H. (2024). Nature-based solutions and climate resilience: a bibliographic perspective through science mapping analysis. Buildings 14:1492. doi: 10.3390/buildings14061492

[B106] Vermelho A. B. Da Silva Cardoso V. Domingos L. T. S. Akamine I. T. Amenu B. Osei B. K. . (2025). Advancements in microbial applications for sustainable food production. Foods 14:3427. doi: 10.3390/foods14193427 41097595 PMC12523703

[B107] Viana C. M. Freire D. Abrantes P. Rocha J. Pereira P. (2021). Agricultural land systems importance for supporting food security and sustainable development goals: a systematic review. Sci. Total Environ. 806(Pt 3):150718. doi: 10.1016/j.scitotenv.2021.150718 34606855

[B108] Wang A. Fu W. Feng Y. Liu Z. Song D. (2022). Synergetic effects of microbial-phytoremediation reshape microbial communities and improve degradation of petroleum contaminants. J. Hazard. Mater. 429:128396. doi: 10.1016/j.jhazmat.2022.128396 35236043

[B109] Wang K. Zhou L. Meng S. Wang Y. Yu B. Wang J. (2023). Anaerobic membrane bioreactor for real antibiotic pharmaceutical wastewater treatment: positive effect of fouling layer on antibiotics and antibiotic resistance genes removals. J. Clean. Prod. 409:137234. doi: 10.1016/j.jclepro.2023.137234

[B110] Wang S. Ma X. Liu Y. Yi X. Du G. Li J. (2020). Fate of antibiotics, antibiotic-resistant bacteria, and cell-free antibiotic-resistant genes in full-scale membrane bioreactor wastewater treatment plants. Bioresour. Technol. 302:122825. doi: 10.1016/j.biortech.2020.122825 31986335

[B111] Wang X. Chi Y. Song S. (2024). Important soil microbiota's effects on plants and soils: a comprehensive 30-year systematic literature review. Front. Microbiol. 15:1347745. doi: 10.3389/fmicb.2024.1347745 38591030 PMC10999704

[B112] Wu J. Han Z. Ma X. Su M. Hamidian A. H. Zhang Y. . (2025). A database on antibiotics and antibiotic resistance in wastewater and solid waste from pharmaceutical industry based on a systematic review. China CDC Weekly 7, 92–100. doi: 10.46234/ccdcw2025.015 39867820 PMC11757902

[B113] Youssef Y. A. Abuarab M. E. Mahrous A. Mahmoud M. (2023). Enhanced degradation of ibuprofen in an integrated constructed wetland-microbial fuel cell: treatment efficiency, electrochemical characterization, and microbial community dynamics. RSC Adv. 13, 29809–29818. doi: 10.1039/D3RA05729A 37829716 PMC10566547

[B114] Yuan W. Liu Y. Shang Y. Bai M. Li L. Li X. . (2025). Fate and removal of oxytetracycline and antibiotic resistance genes in vertical-flow constructed wetland with different substrates. Water 17:1412. doi: 10.3390/w17101412

[B115] Zackariah G. S. K. Tremblay L. A. Li Z. Palmer B. Liu X. An S. . (2025). Antibiotics, antibiotic-resistant bacteria, and genes in agri-foods: a global review of the consumption risks to human health. Integr. Environ. Assess. Manag. 21, 1255–1280. doi: 10.1093/inteam/vjaf084 40580470

[B116] Zhang H. Jiang H. Yin J. Wang T. Zeng W. Lin D. . (2025). Biofilm response in potential difference-enhanced membrane-aerated biofilm systems for accelerated antibiotic removal and ARG mitigation. Water Res. 285:124154. doi: 10.1016/j.watres.2025.124154 40644847

[B117] Zhang Q.-Y. Zhang Y.-Y. Liu J.-S. Li X.-C. Zhu Z.-L. Feng X.-Y. . (2025). Integrating One Health governance in China: assessing structural implementation and operational entry points. One *Health* 21:101209. doi: 10.1016/j.onehlt.2025.101209 PMC12495039 41049402

[B118] Zhang R.-M. Chen X.-J. Li Y.-F. Tan H.-Z. Huang W.-Q. Li L.-L. . (2025). Removal of antibiotic resistance from wastewater in aquatic ecosystems dominated by submerged macrophytes. J. Hazard. Mater. 489:137706. doi: 10.1016/j.jhazmat.2025.137706 40010218

[B119] Zheng D. Yin G. Liu M. Chen C. Jiang Y. Hou L. . (2021). A systematic review of antibiotics and antibiotic resistance genes in estuarine and coastal environments. Sci. Total Environ. 777:146009. doi: 10.1016/j.scitotenv.2021.146009 33676219

[B120] Zheng S. Wang Y. Chen C. Zhou X. Liu Y. Yang J. . (2022). Current progress in natural degradation and enhanced removal techniques of antibiotics in the environment: a review. Int. J. Environ. Res. Public Health 19:10919. doi: 10.3390/ijerph191710919 36078629 PMC9518397

[B121] Zhou Z. Cui E. Abid A. A. Zhu L. Xu J. Chen H. (2024). Evaluating the impact of biochar amendment on antibiotic resistance genes and microbiome dynamics in soil, rhizosphere, and endosphere at field scale. J. Hazard. Mater. 477:135440. doi: 10.1016/j.jhazmat.2024.135440 39111179

